# Exosome-drug conjugates delivery: a promising strategy for ameliorating the pharmacokinetic profile of artesunate

**DOI:** 10.3389/fbioe.2024.1437787

**Published:** 2024-08-12

**Authors:** Da Wang, Yunfei Bai, Guogang Cheng, Shengqiang Shen, Gengwu Xiao, Demei Ma, Ganggang Zhao, Wei Chen, Tianshi Li, Litao Zhang, Xiaohu Ge

**Affiliations:** ^1^ TINGO Exosomes Technology Co., Ltd., Tianjin, China; ^2^ Exosome Origin (Shenzhen) Technology Co., Ltd., Shenzhen, China; ^3^ Plastic & Cosmetic Surgery, Peking University Shenzhen Hospital, Shenzhen, Guangdong, China; ^4^ Department of Dermatology, Tianjin Academy of Traditional Chinese Medicine Affiliated Hospital, Tianjin, China

**Keywords:** exosomes, artesunate, bio-conjugation, delivery, pharmacokinetic

## Abstract

Artesunate (ATS) is considered the most widely employed artemisnin derivative in the treatment of *Plasmodium falciparum* malaria. However, poor solubility and low bioavailability of ATS limit its further clinical application. Herein, we developed a new strategy based on the exosome (exo) - drug conjugation (EDC) using the milk-derived exosomes for ATS delivery. The Exo-ATS conjugates (EACs) which formed via a facile bio-conjugation of ATS to the exosomal surface, have been demonstrated to be able to not only boost the solubility and bioavailability of ATS but also enable a sustained-release of ATS from exosomes. Maximal improvement of 71.4-fold in the solubility of ATS was attained by EACs. The corresponding entrapment efficiency and drug loading capacities were found to be 90.3% and 73.9% for EACs, respectively. Further, *in vivo* pharmacokinetics study manifested that maximum 2.6-fold improved bioavailability of ATS was achieved by oral delivery of EACs. Moreover, EACs displayed a distinct sustained-release profile of maximum 36.2-fold prolonged half-life of ATS via intravenous delivery. We reported that for the first time the administration of EACs could be a potential drug delivery strategy aimed at ameliorating the pharmacokinetic profile of ATS based on our encouraging results and hoped that our work opened up a new avenue for the development of EDC delivery system.

## 1 Introduction

Artemisinin is widely identified as a potent and commonly used agent against *P. falciparum* malaria, which was first discovered from the Chinese medicinal plant *Artemisia annua L.* in 1972 by Youyou Tu (2015 Nobel Prize in Physiology or Medicine) (Tu, 1999; Tu, 2011; Tu, 2016). As the first-line antimalarial agents, artemisinin and its derivatives have recently attracted increasing attention and formed the foundation of the global clinical treatment for *Plasmodium falciparum* malaria (Morris et al., 2011; Tu, 2011; Duan et al., 2019). Of the semi-synthetic derivatives of artemisinin, artesunate (ATS) is considered the most potent and promising agent against *P. falciparum* malaria attribute to the low toxicity and high efficacy for clinical use (Morris et al., 2011; Yang et al., 2021). As with the parent drug artemisinin, ATS is featured with a rare framework of sesquitepene lactone bearing an endoperoxide bridge, which generates up a variety of reactive oxygen species (ROS) such as superoxide anions and carbon-centered free radicals, exhibiting strong antimalarial activity and resulting in parasite death for *P. falciparum* (Gopalakrishnan and Kumar, 2015; Yang et al., 2021). ATS is rapidly degraded into its active metabolite dihydroartemisinin (DART) through hydrolysis under physiological conditions (Morris et al., 2011; Chinaeke et al., 2015; Zhang et al., 2018; Rahman et al., 2020). Compared with artemisinin, ATS shows a slightly increased aqueous solubility ascribed to the introduction of the hemi-succinate group (Yang et al., 2021). Such slightly better solubility however does not render ATS sufficient intestinal absorption, resulting in hardly enhanced bioavailability (Tran et al., 2015; Wang et al., 2021). This hindered the further formulation of ATS as oral administration. Consequently, developing reliable and efficient approaches to improve its bioavailability holds the key to efficient utilization of ATS as oral preparation.

Being a major part of the extracellular vesicles (EVs) secreted by cells, exosomes (Exos) have been widely employed as endogenous nano-scaled (30–150 nm diameter) carriers for oral drug delivery (Théry et al., 2002; Munagala et al., 2016; Kandimalla et al., 2021). As a natural drug vector, exosomes mainly take the advantages of good gastrointestinal stability and potential physiological-barrier permeability for oral drug transportation (Katakowski and Chopp, 2016; Liao et al., 2019). The exosomal membrane consists of a phospholipid bilayer studded with a variety of transmembrane proteins, which is able to protect the drug payload in the lumen of exosomes against degradation under gastrointestinal conditions (Katakowski and Chopp, 2016; Agrawal et al., 2017; Liao et al., 2019; Kalluri and LeBleu, 2020). In addition, it was reported that exosomes inherently have the potential to penetrate the physiological-barriers including the gastrointestinal barrier and blood-brain barrier (Katakowski and Chopp, 2016; Zheng et al., 2019). However, the insufficient production of cell-derived exosomes hardly reaches the requirement for the further pharmaceutical application, resulting in the bottleneck for clinical use of exosomes as drug carriers (Lu and Huang, 2020; de Castilla et al., 2021; Zhong et al., 2021). In comparison, scalable and cost-effective production of exosomes is available from bovine milk (Munagala et al., 2016; Kim et al., 2020; Sanwlani et al., 2020). In addition to the aforementioned good gastrointestinal stability and permeability, milk-derived exosomes displayed satisfactory bio-compatibility in drug delivery. Since bovine milk as one of safe foods supplies important nutritional benefits for human, the milk-derived exosomes were mostly accepted as bio-compatible drug carriers without immune responses for oral administration (Munagala et al., 2016; Zhong et al., 2021; del Pozo-Acebo et al., 2021). In view of these unique advantages, the exosomes derived from bovine milk have recently attracted increasing attention and have been widely considered as the most promising EV-based nano-carriers for delivering oral chemotherapeutic agents. Till today, a variety of small-molecule chemotherapeutic drugs including paclitaxel (PTX) and doxorubicin (DOX) were successfully delivered across the gastrointestinal barrier using milk-derived exosomal carriers (Kim et al., 2016; Munagala et al., 2016; Agrawal et al., 2017). These small-molecule drug payloads were generally loaded into exosomes via various approaches, such as co-incubation, lipofectamine and electroporation (Saari et al., 2015; Agrawal et al., 2017; Sanwlani et al., 2020). Nevertheless, the loading efficiency below 40% is still far from satisfactory, which severely limits the further utilization of milk-derived exosomes for oral drug delivery (Zhong et al., 2021).

**FIGURE 1:**
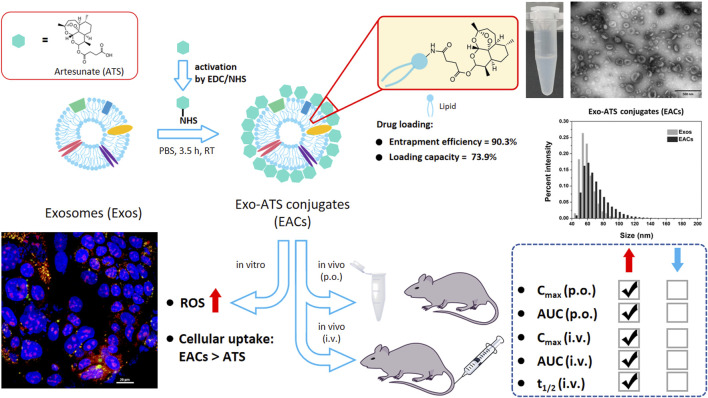
Schematic representation of exosome-drug conjugation (EDC) strategy for ameliorating the pharmacokinetic profile of artesunate (ATS). Exo-ATS conjugates (EACs) were first synthesized by bio-conjugation of ATS to the surface of exosomes via EDC/NHS activation, followed by a series of systematical characterizations including morphology, size distribution, entrapment efficiency and ATS loading capacities. Secondly, cellular uptake and ROS production of EACs were profiled using Caco-2 cells *in vitro*. Further, the pharmacokinetic study *in vivo* of ATS from the EACs was conducted in comparison with free ATS at the same dosage of 7.5 mg/kg using Sprague-Dawley rats via p. o./i.v. delivery.

In this study, we have developed a simple and feasible approach to achieve efficient ATS-loading to the milk-derived exosomes via bio-conjugation of ATS to the surface of the exosomal membranes (Figure 1). The critical quality attributes required for an exosome-based drug vehicle have thus been characterized by a series of techniques, including TEM, NanoFCM as well as UPLC-SEC. The enhancement in solubility of ATS was assessed by determining the conjugated ATS in Exo-ATS conjugates (EACs) via HPLC system. In addition, the cellular uptake of EACs *in vitro* by Caco-2 cells was analyzed by flow cytometry and confocal laser scanning microscopy (CLSM). The inhibition of EACs on proliferation of both of MCF-7 and A549 tumor cells was confirmed as well. Next, the *in vitro* release of ATS from the EACs has been profiled in rat plasma. Further, the amelioration in pharmacokinetic profile *in vivo* of ATS by EACs was evaluated with Sprague-Dawley rats via both oral and intravenous administration in comparison with free ATS. The simple and common bio-conjugation approach used in this work has yet been widely employed for loading insoluble drugs to milk-derived exosomes aimed at improving the drug delivery efficiency. Herein we reported that for the first time the exosome-drug conjugation (EDC) represented a promising strategy for efficiently delivering the drug candidates with poor solubility, low bioavailability and short half-life.

## 2 Materials and methods

### 2.1 Materials

Artesunate (ATS) and dihydroartemisinin (DART) were obtained from Aladdin Industrial Co., Ltd, China. N-(3-(Dimethylamino)propyl)-N′-ethylcarbodiimide hydrochloride (EDC·HCl), N-hydroxysuccinimide (NHS) and dihydroethidium (DHE) were purchased from Sigma-Aldrich Chemical Co. (St. Louis, MO). 2-(4,4-difluoro-5-methyl-4-bora-3a,4a-diaza-s-indacene-3-dodecanoyl)-1-hexadecaoyl-sn-glycero-3-phosphocholine (BODIPY) was purchased from Shanghai ChemeGen Biotech Co., Ltd, China. Anti-CD9 antibody, Anti-TSG101 antibody and Anti-CD81 antibody were all acquired from Beijing Solarbio Science & Technology Co., Ltd, China. BCA Protein Assay Reagent was bought from Fisher Scientific (Pittsburgh, PA). All solvents and reagents used in this work were HPLC analytical grade and were from Sigma Chemical (St. Louis, MO).

### 2.2 Isolation of milk-derived exosomes

Milk-derived exosomes were isolated from raw milk using differential ultra-centrifugation (UC) as previously described with a slight modification (Munagala et al., 2016). Briefly, raw milk was first centrifuged at 15,000 × g for 30 min at 4°C in order to well remove the majority of impurities including fat and casein aggregates (Allegra X-15R Centrifuge, Beckman Coulter). The resulting whey was then collected and subsequently ultra-centrifuged at 100,000 × g for 90 min at 4°C by Optima L-80 XP ultra-centrifuge with rotor SW32Ti (Beckman Coulter). The final supernatant was ultra-centrifuged at 135,000 × g for 2 h at 4°C to pellet exosomes using Optima L-80 XP ultra-centrifuge with rotor SW32Ti (Beckman Coulter). The exosomal pellet was twice washed with PBS buffer and then again concentrated via ultra-centrifugation at 135,000 × g for 2 h at 4°C as aforementioned. Finally, the exosomal pellet was re-suspended in 1 mL PBS buffer followed by filtration through a 0.22 μm Steritop filter for sterilization. The total protein content in the suspension of milk-derived exosomes was quantified using the BCA protein assay and diluted into 1.33 mg/mL prior to storage at −80°C. In this work, we processed 100 L of raw milk at a time in total and finally obtained 5 L milk-derived exosomes (1.33 mg/mL) with a stable yield of approximately 5%.

### 2.3 Synthesis of artesunate NHS ester (ATS-NHS) and preparation of Exo-ATS conjugates (EACs)

A N-hydroxysuccinimide ester of artesunate (ATS) was synthesized by directly activation of ATS using both NHS and EDC. Briefly, artesunate (100.0 mg, 0.26 mmol) was added into the solution of EDC·HCl (65.0 mg, 0.34 mmol) and NHS (39.0 mg, 0.34 mmol) in CH_2_Cl_2_ (3 mL) and the mixed solution was then stirred for overnight at room temperature. The reaction was monitored by TLC (DCM/EtOAc = 5:1, v/v). Concentration under reduced pressure followed by purification through flash column chromatography (DCM/EtOAc = 15:1, v/v) afforded artesunate NHS ester (ATS-NHS, 119.0 mg, 95%). ^1^H NMR (600 MHz, DMSO-*d*_6_) δ 5.69 (d, J = 9.8 Hz, 1H), 5.58 (s, 1H), 3.00 – 2.95 (m, 2H), 2.84 – 2.75 (m, 6H), 2.34 – 2.25 (m, 1H), 2.18 (ddd, J = 14.5, 13.4, 4.0 Hz, 1H), 2.03 – 1.96 (m, 1H), 1.85 – 1.78 (m, 1H), 1.66 – 1.58 (m, 2H), 1.58 – 1.52 (m, 1H), 1.46 (ddt, J = 28.9, 16.1, 7.0 Hz, 2H), 1.38 – 1.29 (m, 1H), 1.29 (s, 3H), 1.21 – 1.14 (m, 2H), 1.01 – 0.90 (m, 1H), 0.89 (d, J = 6.4 Hz, 3H), 0.76 (d, J = 7.1 Hz, 3H). ESI-MS [M + NH_4_]^+^ 499.42.

Afterwards, a series of EACs were prepared according to the formulation shown in Table 1. Briefly, the ATS-NHS solution in DMSO (36 mM) with various volumes of 30, 60, 120 and 250 μL was mixed with 750 μL milk-derived exosomes (Exos) with a concentration of 10^12^ particles/mL, respectively. In each case, PBS buffer (pH 7.4) was added up to a total volume of 1.0 mL and the mixture was then incubated for 3.5 h at room temperature. The produced EACs were eventually purified by column chromatography.

**TABLE 1:** Formulation of the studied Exo-ATS conjugates (EACs).

Batch	Addition of ATS-NHS^a^ solution (μL)	Addition of PBS buffer (μL)	Volume of exos^b^ (μL)
EAC-1	30	220	750
EAC-2	60	190	750
EAC-3	120	130	750
EAC-4	250	0	750

^a^ ATS-NHS = N-hydroxysuccinimide ester of artesunate.

^b^ Exos = milk-derived exosomes.

### 2.4 Qualitative characterization of Exos and EACs

#### 2.4.1 Transmission electron microscopy (TEM)

The samples of Exos and EACs were diluted with a ratio of 1–10 using PBS buffer. 10 μL of samples were then mixed with 10 μL 4% paraformaldehyde (PFA) for 15 min. The fixed samples were individually added onto the 200 mesh grids and incubated for 3 min at 4°C. Subsequently, the samples on the grids were negatively stained with 2% aqueous uranyl acetate for 1 min and dried out in darkness for 15 min. The surface morphology of the samples of both Exos and EACs were analyzed by TEM (Hitachi, HT7800) at 80 kV and AMT XR81B CCD-camera.

#### 2.4.2 Nano-Flow Cytometry analysis (NanoFCM)

In this work particle size distribution of the samples of Exos and EACs was analyzed using the instrument of Nano-Flow Cytometry (NanoFCM), which enables the size characterization of single exosome as small as 40 nm (Tian et al., 2020). Moreover the particle concentrations of Exos and EACs were determined by NanoFCM as well. Herein, the prepared EACs and unmodified exosomes were diluted with a ratio of 1:1000 (v/v) by PBS buffer prior to the measurement. A set of monodisperse silica nanoparticles of four different diameters of 68 ± 2 nm, 91 ± 3 nm, 113 ± 3 nm and 155 ± 3 nm were used to calibrate the particle size. In addition, counting calibration was performed using 250 ± 5 nm monodisperse FluoSpheres of known particle concentration. All the samples were measured by the side scatter (SSC) detector (bandpass filter: FF01-524/24) with an attenuation coefficient of 10% at a sample flow rate of 25.10 nL/min under 1.0 kPa sample flow pressure.

#### 2.4.3 Western blot analysis (WB)

Western blotting for analysis of TSG101, CD9 and CD81, which were regarded as main exosomal surface markers, was carried out to identify the isolated milk-derived exosomes and the prepared EACs (Saari et al., 2015). In brief, the samples of Exos and EACs were suspended into radio immunoprecipitation assay (RIPA) buffer for collecting the proteins, followed by the determination of the total protein content in the samples via BCA assay. Concurrently, the acquired proteins were fractionated with SDS-PAGE electrophoresis, transferred to PVDF membranes and then incubated with primary antibodies: Anti-CD9 (1:1000), Anti-CD81 (1:1000) and Anti-TSG101 (1:10000) at 4°C overnight. Following the incubation of appropriate secondary antibodies, enhanced chemiluminescence (ECL) reagent was added to visualize the protein bands.

#### 2.4.4 Zeta-potential (ZP)

To evaluate the influence of ATS-based bio-conjugation on the surface charge of the milk-derived exosomes, the zeta-potential values of the samples of Exos and EACs were measured in triplicates by ZetaPALS (Brookhaven Instruments). All the samples were diluted in a certain volume of 400 μL of PBS buffer and afterwards the particle concentrations from the samples were analyzed using NanoFCM as above mentioned prior to the measurement.

#### 2.4.5 UPLC-SEC identification of Exos and EACs

The identification of Exos and EACs was conducted by the application of ultra-high performance liquid chromatography-size exclusion chromatography (UPLC-SEC). The separation of the samples was carried out at a flow rate of 0.3 mL/min with 20 mM phosphate buffer (pH 7.2) as liquid phase consisting of 150 mM chloride sodium with a 7.8 × 150 mm Acclaim SEC-1000 LC column (particle size 7 μm, pore size 1000 Å). The presence of the Exos in the eluted fractions was identified by the exosomal protein components, which was detected at 280 nm by UV-absorbance. The ATS from the samples of EACs was monitored at 210 nm in which the ATS conjugated to the EACs was identified at the same retention time of the protein components of EACs.

### 2.5 Quantitative characterization of EACs

#### 2.5.1 HPLC quantification of ATS present in EACs

The ATS coupled to the EACs was quantified by HPLC system with a 4.6 × 250 mm Symmetry C18 column (particle size 5 μm) (Waters). Acetonitrile and phosphate buffer (pH 3.0) (44:56, v/v) were employed as liquid phase for separation. The ATS, with a retention time of 19.6 min, was spectro-photometrically detected at 216 nm and the quantification of total ATS was performed against a standard curve of ATS. Each batch of the prepared EACs as described above (Section 2.3) was divided into two equal parts (Part I and II). Part I (control) was directly analyzed using HPLC system, while Part II was treated with protease at 37°C for 6 h to completely release the conjugated ATS molecules prior to the HPLC measurements. The detected ATS from Part I corresponded to the residue of free ATS in EACs. The content of the conjugated ATS in EACs was thus gained by subtracting the quantity of free ATS in Part I from the detected total amount of ATS in Part II.

#### 2.5.2 Entrapment efficiency

Despite the distinct difference between the bio-conjugation and encapsulation techniques, both of them are capable of loading ATS to the exosome-based cargoes (Jin et al., 2013; Chinaeke et al., 2015; Chen et al., 2019). Therefore, in this work we employed ‘entrapment efficiency’ to substitute for encapsulation efficiency, in order to more accurately evaluate the effect of bio-conjugation. The entrapment efficiency (EE, %) was calculated using the following formula:

$$EE\left(\%\right)=\frac{M_{2}-M_{1}}{M_{0}}\times 100$$ (1)

Where *M*_*1*_ and *M*_*2*_ represent the detected content of ATS in Part I and II of each batch of EACs, respectively. *M*_*0*_ refers to the initial addition of ATS in either Part I or II, which is equal to half of the initial amount of added ATS for each batch of EACs.

#### 2.5.3 Drug loading capacities

Drug loading capacities (LC) was widely used for assess the capacity of nano-carriers for drug loading (Chinaeke et al., 2015). Herein, LC referred to the ratio between the content of the conjugated ATS and the total exosomal proteins for each batch of EACs, which was determined by:

LC mg ATS/100 mg Exo−protein=M2−M1MExos×100 (2)

Where *M*_*1*_ and *M*_*2*_ stand for the detected content of ATS in Part I and II of each batch of EACs, respectively. *M*_*Exos*_ refers to the content of exosomal proteins in either Part I or II, which is equal to half of the amount of total exosomal proteins in each batch of EACs.

#### 2.5.4 Drug-to-exosome ratio (DER)

For better understanding the EACs and the EDC delivery system, a new concept of drug-to-exosome ratio (DER) was introduced for the first time in our work. The term ‘DER’ was employed to illustrate that how many drug molecules stood on the surface of the exosomes by bio-conjugation. The calculation formula of DER was as follows:

DER molecules per Exo−particle=M2−M1384.42×NANO (3)

Where *M*_*1*_ and *M*_*2*_ represent the detected content of ATS in Part I and II of each batch of EACs, respectively. *N*_*A*_ refers to Avogadro constant, and *N*_*0*_ the mean number of exosomal particles in either Part I or II, which is equal to half of the total quantity of exosomal particles in each batch of EACs.

### 2.6 Stability study

Each batch of the prepared EACs (Section 2.3) was stored at 4°C for up to 7 days. Size distribution, zeta-potential and LC of each batch of EACs were examined daily as described in 2.4.2, 2.4.4 and 2.5.3, respectively. Further, the long-term (6-month) stability of EACs was investigated under various storage conditions. The EACs were stored at 4°C, −20°C and −80°C, respectively. Moreover, the freeze-dried powder of the EACs obtained using lyophilization was stored at room temperature. The EACs were lyophilized in this work as follows: both of trehalose and mannitol were chosen as cryoprotectants and dissolved into EACs to obtain a final concentration of 5% (w/v). Typically 1 mL liquid mixture containing EACs was transferred in a 5 mL-vial and frozen in a freeze dryer (SCIENTZ, Ningbo, China). The purity, size distribution, particle concentration, zeta-potential and LC of the studied EACs were examined after 1-month, 3-month and 6-month, respectively.

### 2.7 Cellular uptake of EACs *in vitro*

The cellular uptake profiles of EACs *in vitro* were investigated using Human colonic adenocarcinoma cell line (Caco-2), which was acquired from the Procell Life Science & Technology Co, Ltd. (Wuhan, China). Caco-2 cells were cultured in 75 cm^2^ cell culture dishes in Dulbecco’s modified Eagle’s medium (DMEM) supplemented with 10% fetal bovine serum (FBS) and 1% penicillin-streptomycin reagent (PS). The culture medium was refreshed every 2–3 days. For the uptake assay of EACs, 5 × 10^4^ Caco-2 cells were seeded in each well of a 24-well plate and cultured in humidified 5% CO_2_ atmosphere at 37°C until 70% confluence was reached.

For the cellular uptake assay, EAC-3 containing 1.5 mg/mL ATS was first labeled with BODIPY fluorescent dye protected from light for 30 min at room temperature and purified by column chromatography as previously described in Section 2.3 to remove the free BODIPY probes. A sample of the unmodified Exos was similarly labeled and purified. Secondly, the fluorescent labeling efficiency of both of the BODIPY-labeled Exos and EAC-3 was analyzed by NanoFCM using 488 nm laser (FITC channel) with an attenuation coefficient of 10% at a sample flow rate of 25.10 nL/min under 1.0 kPa sample flow pressure. Next, the BODIPY-labeled EAC-3 or free ATS (equivalent to 1.5 mg/mL ATS) was added to the Caco-2 cells and further incubated at 37°C for 2 and 4 h. The BODIPY-labeled EAC-3 was diluted with PBS buffer and then added to the culture medium at a final concentration of 50 μg/mL. The free ATS of 1.5 mg/mL was similarly diluted. After incubation, the culture medium was removed. Then the Caco-2 cells were twice rinsed by DPBS, followed by fixation with 4% PFA for 15 min and then staining for 5 min using 4’,6-diamidino-2-phenylindole (DAPI). The fluorescence images were recorded using an A1 HD25 (Nikon) confocal laser scanning microscopy (CLSM) and an eclipse *Ti*2 wide field microscope with respective ×60oil ommersion objective.

At 0, 2 and 4 h, the fluorescent signals from the collected Caco-2 cells were analyzed by flow cytometry (Gallios, Beckman Coulter) using FITC channel (488 nm laser). The geometrical mean fluorescence intensity (GMFI) of the Caco-2 cells was determined as well.

Moreover the uptake efficiency of EAC-3 and free ATS (equivalent to 1.5 mg/mL ATS) by the Caco-2 cells was also measured. After incubation, the cells were twice washed by DPBS and then lysed with cell lysate, followed by centrifuged at 12,000 × g for 5 min at 4°C. Afterwards, the supernatant was collected for analyse at 216 nm using HPLC system.

### 2.8 ROS production of EACs *in vitro*

The ROS production of EACs after uptake by the Caco-2 cells was profiled using dihydroethidium (DHE) method with slight modifications (Chen et al., 2022). In brief, Caco-2cells were cultured as described in section 2.7, followed by further incubation with Exos, free ATS and BODIPY-labeled EAC-3 (section 2.7) for 4 h, respectively. Then the culture medium was discarded and 0.1 mM DHE reagent was added to the cells, followed by incubation for 15 min at 37°C. After incubation, the Caco-2 cells were detached by trypsin treatment and collected after several washes by DPBS. Following this, the geometrical mean fluorescence intensity (GMFI) of the yielded ethidium due to ROS production for each group was determined by flow cytometry. The corresponding fluorescence images were recorded by confocal laser scanning microscopy (CLSM) as described in section 2.7. The ROS generation of EACs was evaluated by relative ROS level, which was determined as follows:

$$\mathrm{RelativeROS level}\left(\%\right)=\frac{GMFI\left(EACs\right)-GMFI\left(control\right)}{GMFI\left(ATS\right)-GMFI\left(control\right)}\times 100$$ (4)

Where *GMFI*(EACs), *GMFI*(ATS) and *GMFI*(control) stand for the detected GMFI of EACs, ATS and control (saline) groups, respectively. All the samples were measured in triplicate in this study.

### 2.9 Inhibition of EACs on cell proliferation

The effect of each batch of the prepared EACs (Section 2.3) on cell proliferation was evaluated using MCF-7 human breast cancer cells and A549 human lung cancer cells by standard methyl thiazolyl tetrazolium (MTT) assay. In brief, 5 × 10^3^ MCF-7 or A549 cells were seeded in each well of a 96-well plate and cultured in humidified 5% CO_2_ atmosphere at 37 °C for 24 h. Subsequently, the culture medium was discarded and replaced by free ATS or EACs with varying concentration of ATS, followed by further incubation for 48 h. Afterwards, 10 μL MTT reagent (5 mg/mL) was added to each well and co-incubated for another 4 h. Then all culture medium for each well were removed and replaced by 150 μL DMSO. After 10 min vibration of the plates, the viable cells were detected by measuring the absorbance at 570 nm using a microplate reader (FLUOstar Omega, BMG Labtech). The untreated cells worked as negative control (NC). The cell viability of each group was expressed as a percentage relative to that of NC. Tests were performed in triplicate at each ATS concentration. The same procedure was used for assessing the effect of the unmodified exosomes derived from milk against the aforementioned cell lines.

### 2.10 *In vitro* release of ATS from EACs and the degradation of ATS

The study with respect to *in vitro* release of ATS from EACs was conducted by quantitative determination of ATS in rat plasma by acquity UPLC system using a 2.1 × 50 mm BEH C18 column (particle size 1.7 μm) (Waters). Verapamil was employed as internal standard for determining ATS concentration in rat plasma in this study. The separation was carried out with a flow rate of 0.3 mL/min using a gradient liquid phase consisting of 5% (v/v) acetonitrile-95% (v/v) water as mobile phase A containing 0.1% (v/v) formic acid and acetonitrile as mobile phase B containing 0.1% (v/v) formic acid. The gradient was formulated as Table 2:

**TABLE 2:** Formulation of the used gradient liquid phase for determination of artesunate (ATS) in rat plasma.

Time (min)	Mobile phase A (%)	Mobile phase B (%)
0.00	80	20
0.50	80	20
1.50	5	95
2.50	5	95
2.60	80	20
4.00	80	20

The retention time of ATS, was 2.06 min compared with 1.88 min of verapamil, which was monitored at 210 nm. In order to investigate the *in vitro* release of ATS, from EACs into rat plasma; EAC-1, EAC-2 and EAC-3 (equivalent to 0.1, 0.5 and 1.5 mg/mL ATS, respectively) were mixed with rat plasma with a ratio of 1:49 (v/v) in the presence of EDTA-K_2_, anti-coagulation at 37°C. The rat plasma-ATS, concentration of each batch of EACs was determined by UPLC, at 0, 2, 4, 6, 8, 10 and 12 h, respectively. At each sampling point, 50 μL cold MeOH, was added to 50 μL plasma sample to precipitate the exosomal proteins, followed by the introduction of 100 μL verapamil working solution into the supernatant. After mixing for 3 min by vortex mixer, the mixture was centrifuged at 3,000 × g at 4°C for 15 min. Thus, 100 μL of the supernatant was mixed with 100 μL MilliQ water, followed by centrifugation at 3,000 × g at 4°C for another 5 min 5 μL of the resulted supernatant was analyzed by the UPLC, method as aforementioned. The rat plasma-ATS, concentration was calculated against a standard curve of ATS, which was obtained using a series of standard plasma samples with varying concentrations of ATS., herein, the release rate of ATS, from EACs, which was defined as the ratio of the content of the free ATS, in plasma at a certain time to the total content of ATS, present in EACs, was utilized to evaluate the *in vitro* release level of ATS., The release rate of ATS, was calculated via the following formula.

$$Release\;rate\left(\%\right)=\frac{{Conc}_{t}\left(plasma-ATS\right)\times V\left(plasma\right)}{m\left(ATS\;in\;EACs\right)}\times 100$$ (5)

Where *Conc*_*t*_ (plasma-ATS) represents the calculated plasma-ATS concentration at a certain time, and *V* (plasma) the volume of the plasma sample. *m* (ATS in EACs) refers to the total content of the ATS in EACs. All the plasma samples were measured in triplicate in this study.

Considering the rapid degradation of ATS in rat plasma, the concentration of its metabolite dihydroartemisinin (DART) in the plasma samples were determined by UPLC system as well. The details regarding the UPLC quantitative method of DART in rat plasma were provided in Supplementary Material. Herein the release profiles of DART from EAC-3 and free ATS (equivalent to 1.5 mg/mL ATS) was estimated during 12 h by UPLC determination of DART in rat plasma.

### 2.11 Pharmacokinetic (PK) study

All the animal experiments were conducted under the ethical permission 20220512-01 of the Institutional Animal Care and Use Committee (IACUC) of China by YUGEN MedTech Co., Ltd (Tianjin, China) in GLP level animal facility. All the animal experiments can be carried out using neither the models *in vitro* nor lower animals. The minimum use of animals were required for the pharmacokinetic (PK) study and the mice were housed in an air-conditioned room with free access to water and a standard laboratory diet. In this work blood was sampled from jugular vein of mice and all operations were followed with the procedures with respect to blood collection.

The pharmacokinetic (PK) study of EACs was performed with Sprague-Dawley rats (200–230 g, 6–8 weeks old, n = 16) that administrated with EACs in comparison with ATS active pharmaceutical ingredient (API) at same dosage of 7.5 mg/kg, which was sub-divided into four groups: (i)/(ii) intravenous administration (i.v.) of EAC-3 *versus* ATS API (equivalent to 1.5 mg/mL ATS) and (iii)/(iv) oral administration (p.o.) of EAC-3 *versus* ATS API (equivalent to 0.75 mg/mL ATS after dilution). The ATS concentrations in rat plasma (i.v.) were determined by the UPLC method as described in Section 2.8 at 5, 15, 30 min, 1, 2, 4, 6, 9 and 24 h, respectively. While the rat plasma-ATS concentrations (p.o.) were determined at 15, 30 min, 1, 2, 4, 6, 9 and 24 h, respectively. At each sampling point, 0.3 mL blood samples were collected in anti-coagulation tubes with EDTA-K_2_ and placed on ice for 30 min. The plasma samples were thus obtained by centrifugation at 3000 *g* at 4°C for 5 min and stored at −80°C prior to determination. A series of pharmacokinetic parameters including peak time (T_max_), peak concentration (C_max_), area under the curve (AUC), half-life period (t_1/2_), clearance (CL), apparent volume of distribution (V_d_) and mean residence time (MRT) were calculated using non-compartmental analysis (Phoenix WinNonlin software version 8.1.0, Pharsight, United States) according to the drug plasma concentration-time curves with respect to ATS. Considering the rapid degradation of ATS in rat plasma, the concentration of its metabolite dihydroartemisinin (DART) in all the plasma samples were determined by UPLC system as well. The details regarding the UPLC quantitative method of DART in rat plasma were provided in Supplementary Material.

### 2.12 Statistical analysis

Bars represented the mean ± SD from three independent experiments. GraphPad Prism 5 (Graph Pad, SanDiego, CA, United States) was employed for the statistical analysis. For comparisons of two experimental groups, a Student’s t-test was conducted and the differences in between were considered statistically significant at the 95% confidence level (*p* < 0.05). Values of *p* < 0.05, *p* < 0.01, and *p* < 0.001 were denoted by *, **, and ***, respectively.

## 3 Results

### 3.1 Preparation and qualitative characterization of EACs

**FIGURE 2:**
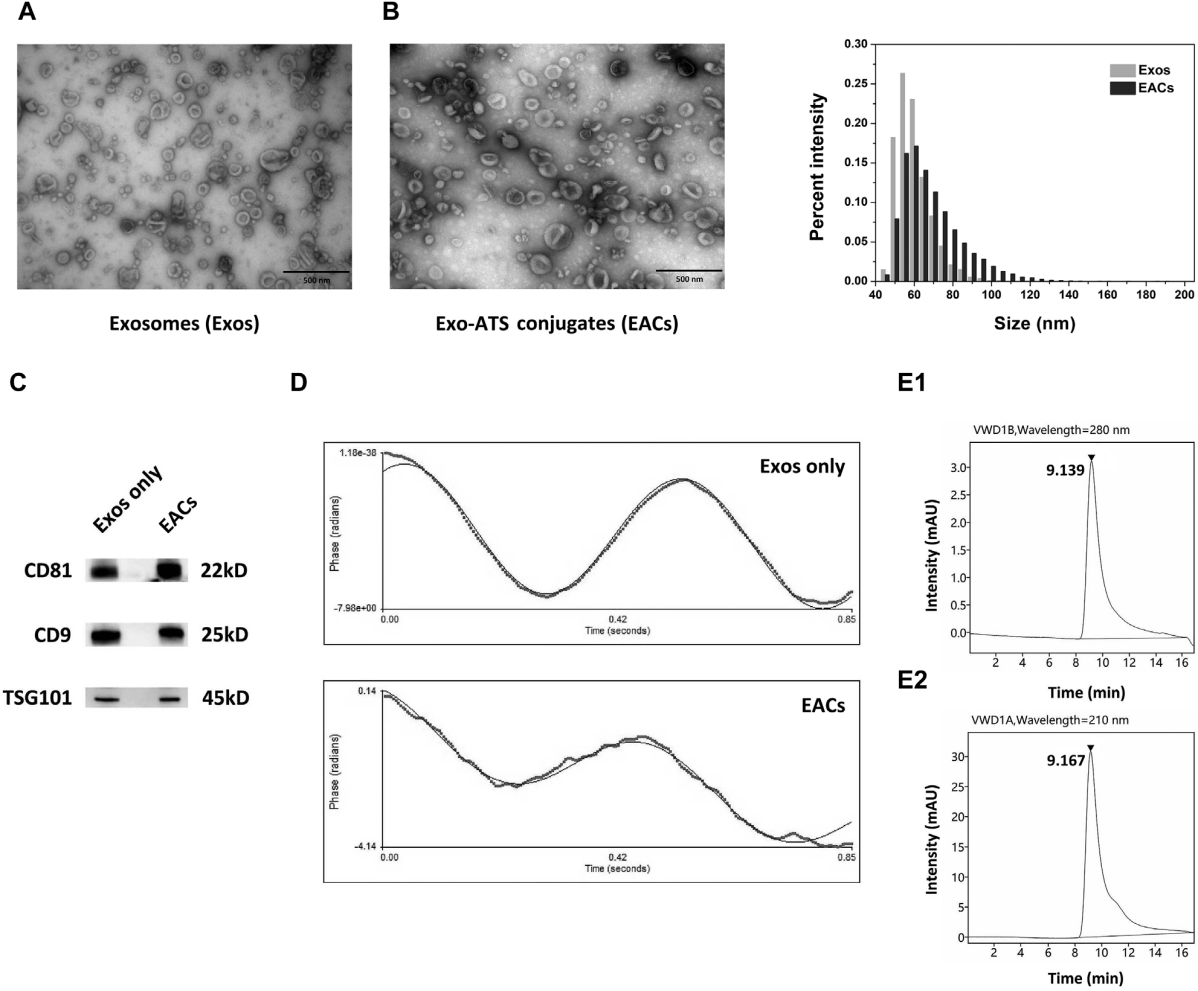
Qualitative characterization of the milk-derived exosomes (Exos) and Exo-ATS conjugates (EACs). **(A)** TEM images of the negatively stained Exos (left) and EACs (right). Scale bar = 500 nm. **(B)** Bar diagram depicting variation in size distribution following the ATS binding to the Exos by NanoFCM analysis. **(C)** Western blot analysis of the main exosomal surface biomarkers of CD81, CD9 and TSG101 of Exos and EACs **(D)** Zeta-potential of the Exos and EACs measured by ZetaPALS. **(E1, E2)** Identification of the EACs detected at 280 nm (top) and 210 nm (bottom) by ultra-high performance liquid chromatography-size exclusion chromatography (UPLC-SEC) determination, respectively. The concentration of ATS in EACs is 1.5 mg/mL. Data were presented as mean ± SD (*n* = 3).

In this work, exosomes (Exos) were isolated from bovine milk using differential ultra-centrifugation (UC) method, followed by a systematical characterization with a range of techniques including TEM, NanoFCM, ZetaPALS, UPLC-SEC and WB analysis (Figure 2). Given the typical saucer-shaped morphology of exosomes shown by TEM (Figure 2A, left) in conjunction with the profile of the common exosomal protein markers of CD81, CD9 and TSG101 by WB analysis (Figure 2C), it was suggested the successful extraction of exosomes from bovine milk (Munagala et al., 2016; Agrawal et al., 2017).

Next, in order to prepare the EACs, the carboxyl group of artesunate (ATS) was first activated in the presence of EDC and NHS, leading to formation of reactive artesunate ester (ATS-NHS) (Figure 1). The structure of ATS-NHS was verified by MS and ^1^H-NMR (Supplementary Figures S1, S2 included in Supplementary Material). Subsequently, ATS was successfully conjugated to the Exos via bio-conjugation chemistry, which was based on the condensation reaction between ATS-NHS and the primary amine groups positioned on the surface of Exos (Xu et al., 2020; Wu et al., 2021). The EACs were thus formed by amide-linkage in PBS buffer in accompany with the withdrawal of the NHS moiety from ATS-NHS. The variation in particle size distribution due to the bio-conjugation of ATS to the Exos has been shown in Figure 2B. A slight shift from 62.59 to 68.74 nm in particle size on average was observed following the formation of EACs as measured by NanoFCM (Table 3). Note that the intact and well saucer-like structure of Exos still remained after bio-conjugation of ATS (Figure 2A, right) and the tested three protein markers were all enriched in the EACs as well (Figure 2C). Such results indicated that the morphology, size distribution and protein markers of the milk-derived exosomes were not significantly affected by the biological coupling of ATS. In addition Figure 2D showed that the zeta-potential of EACs has shifted towards a less negative value of −13.00 mV compared to the unmodified exosomes (−15.63 mV). Nevertheless, the zeta-potential of Exos and EACs were quite similar and thus could be considered to be unaltered during the bio-conjugation. The mild change in zeta-potential was also expected ascribed to the coupled coating of uncharged ATS molecules.

**TABLE 3:** Critical quality attributes of the milk-derived exosomes (Exos) and Exo-ATS conjugates (EACs).

Formulation	Mean size (nm)	Zeta-potential (mV)	Purity (%)
Exos	62.59 ± 9.27^a^	−15.63 ± 0.12	>99.0
EACs	68.74 ± 15.80	−13.00 ± 1.60	>99.0

^a^ Data were presented as mean ± SD (*n* = 3).

For the evaluation of the purity of the Exos and EACs as well as identification of the conjugated ATS in EACs, ultra-high performance liquid chromatography-size exclusion chromatography (UPLC-SEC) was employed in our study. A distinct peak was present at 9.185 min as measured at 280 nm (Supplementary Figure S3), corresponding to the presence of the Exos with a good purity (>99.0%, Table 3). Similarly, as shown in Figure 2E1, the peak representing the EACs was observed at 9.139 min together with a nearly unaltered purity (>99.0%, Table 3), implying a better maintainence of the structural integrity of the milk-derived exosomes. Moreover we identified the ATS in EACs at 210 nm using UPLC-SEC in this study. Figure 2E2 displayed that the retention time of the ATS in EACs was 9.167 min at 210 nm, which was comparable with that of the entire EACs at 280 nm. This directly confirmed a successful bio-conjugation of ATS to the exosomes.

### 3.2 Quantitative characterization of EACs

**FIGURE 3:**
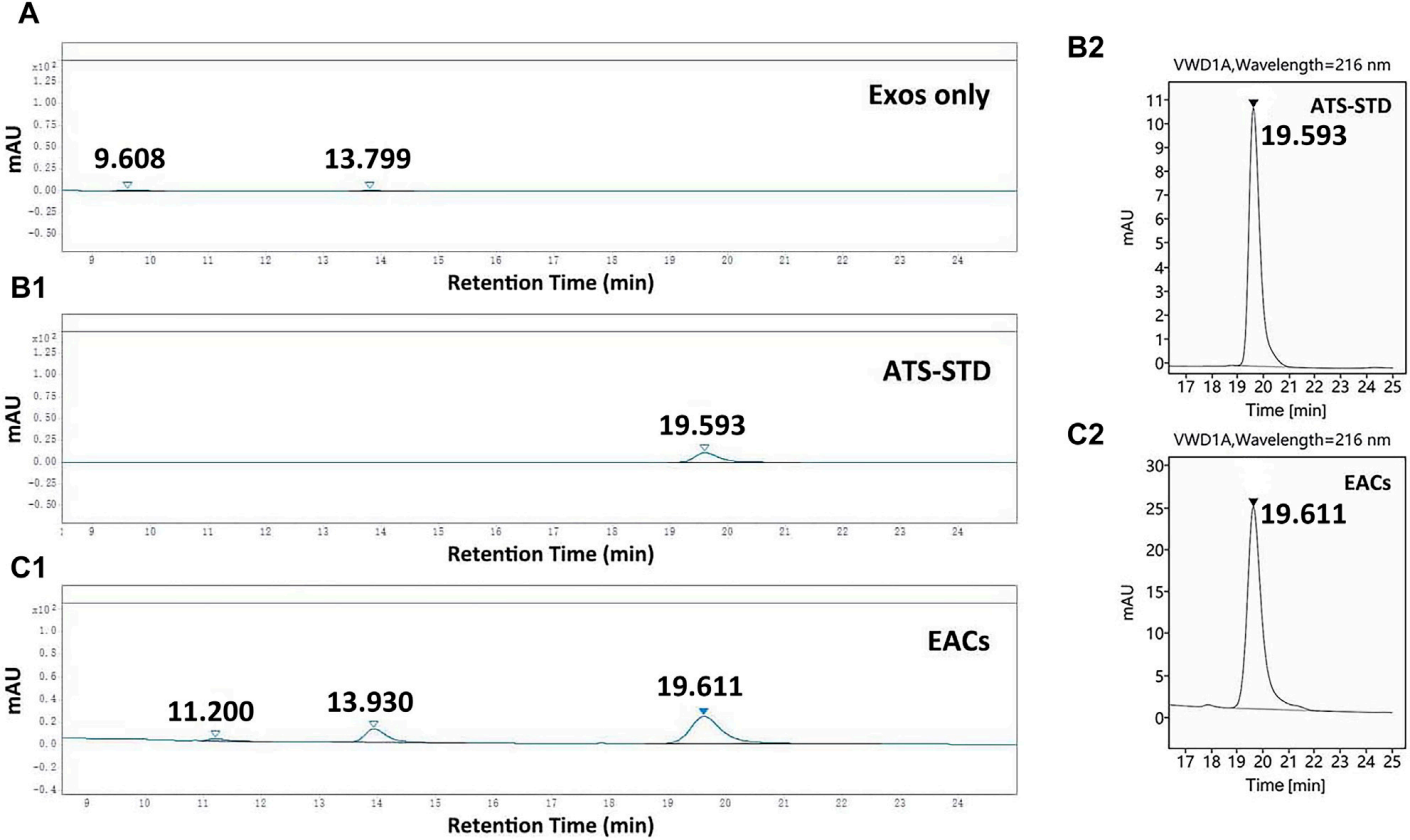
HPLC chromatograms of **(A)** the unmodified exosomes (Exos only), **(B1, B2)** ATS-standard (ATS-STD) and **(C1, C2)** Exo-ATS conjugates (EACs). The retention time of ATS-STD of 0.5 mg/mL was 19.6 min, which was found again in EACs. Two new peaks (retention times 11.2 and 13.9 min) present in EACs likely originated from Exos only. Data were presented as mean ± SD (*n* = 3).

To evaluate the improvement in the solubility of ATS by EACs, the total content of the ATS coupled to the Exos was quantified using HPLC system. A linear curve of the ATS-standard (ATS-STD) for quantification was gained with varying concentrations of ATS range from 0.01 to 1.0 mg/mL. In this work, four different formulations of EACs (Table 1) were prepared and then treated with protease to completely release the conjugated ATS molecules via hydrolysis of the amide-linkage prior the HPLC measurements. The sample of the unmodified exosomes (Exos only) was similarly treated with protease. The content of the conjugated ATS in each batch of EACs was obtained by subtracting the quantity of unconjugated ATS from the detected total amount of ATS. The HPLC quantitative analysis of EACs was displayed in Figure 3.

A flat HPLC chromatogram for the case of Exos only was shown in Figure 3A, indicative for the negative background responses of the unmodified exosomes. The retention time of ATS-STD (0.5 mg/mL) was found to be 19.6 min (Figures 3B1, B2) where a larger peak was again observed in EACs (Figures 3C1, C2). This corresponded to the ATS present in EACs. Moreover no responsiveness regarding ATS was seen for the cases of all the batches of EACs without treatment by protease, which suggested that the free ATS fragment in EACs was negligible (data not shown). Therefore the detected total amount of ATS by HPLC was equal to the content of the conjugated ATS in each batch of EACs, which could be directly quantified by the peak area ratio of ATS-STD to EACs (Table 4).

**TABLE 4:** Quantitative characterizations of the studied Exo-ATS conjugates (EACs).

Characterization	Batch			
	EAC-1	EAC-2	EAC-3	EAC-4
Particle concentration (particles/mL)	1.24 × 10^12^	1.11 × 10^12^	1.36 × 10^12^	1.91 × 10^12^
Total Exo-protein^a^ concentration (mg/mL)	1.88 ± 0.54^b^	1.76 ± 0.25	2.03 ± 0.34	2.22 ± 0.77
Concentration of ATS (mg/mL)	0.12 ± 0.22	0.40 ± 0.33	1.50 ± 0.18	1.40 ± 0.88
Improvement in the solubility of ATS (folds)	5.7	19.0	71.4	66.9
EE^c^ (%)	27.8	48.6	90.3	40.6
LC^d^ (mg ATS/100 mg Exo-protein)	6.4	22.7	73.9	63.1
DER^e^ (molecules per Exo-particle)	1.45 × 10^5^	5.70 × 10^5^	1.72 × 10^6^	1.15 × 10^6^

^a^ Exo-protein = exosomal protein.

^b^ Data were presented as mean ± SD (*n* = 3).

^c^ EE, entrapment efficiency.

^d^ LC, loading capacities.

^e^ DER, drug-to-exosome ratio.

As shown in Table 4, the EACs exhibited high EE (%) of 27.8%–90.3%, showing an efficient bio-conjugation of ATS to the Exos. Moreover we found that the EE (%), LC and DER of EACs positively correlated with the initial addition of ATS-NHS to the Exos and reached their maximum at EAC-3. The general decline in EE (%), LC and DER was seen at EAC-4. Thus, this led to the maximal concentration of 1.5 mg/mL of ATS in EAC-3 in our work, exhibiting the best improved solubility of ATS with approximately 71.4-fold higher than 21 μg/mL of free ATS in PBS buffer (the LC-MS/MS determination of the aqueous solubility of ATS included in Supplementary Material). Accordingly, the highest EE (%) and LC were 90.3% and 73.9%, respectively. The maximal DER of 1.72 × 10^6^ ATS molecules per Exo-particle was obtained by EAC-3 as well. Therefore, the formulation of EAC-3 as the optimal batch of EACs was studied in the following investigation *in vitro* and *in vivo.*

In addition, ATS was loaded to exosomes with the same initial addition of ATS and exosomes as EAC-3 via a variety of conventional physical approaches including co-incubation, electroporation and lipofectamine transfection, respectively, and the corresponding ATS loading capacities (LC) were characterized as well. As seen in Table 5, simple co-incubation, electroporation and lipofectamine transfection resulted in extremely low LC of 1.9%, 1.8% and 1.6%, respectively, indicating an inefficient loading of ATS to exosomes by conventional physical methods. In comparison, the optimization for ATS loading to milk-derived exosome-based cargoes was achieved by EDC strategy.

**TABLE 5:** Quantitative characterizations of artesunate (ATS)-loaded exosomes by co-incubation, electroporation and lipofectamine transfection.

Characterization	Methodology		
	Co-incubation	Electroporation	Lipofectamine transfection
Concentration of ATS (mg/mL)	0.037 ± 0.029^a^	0.035 ± 0.016	0.031 ± 0.018
LC^b^ (mg ATS/100 mg Exo-protein)	1.9	1.8	1.6

^a^ Data were presented as mean ± SD (*n* = 3).

^b^ LC, loading capacity.

### 3.3 Stability study of EACs

Since the storage of EACs at 4°C for approximately 1 week was required for the subsequent studies *in vitro* and *in vivo,* we carried out the stability study of the formulation of EAC-3 under the storage conditions by determining the mean size, zeta-potential and ATS loading capacity during a 7-day observation period (Table 6). A slight variation in mean size of EACs indicated that the integrity of EACs was basically maintained at 4°C throughout the storage period. Furthermore zeta-potential of EACs remained unaltered during 1 week, confirming the good colloidal stability. This was in line with no observation of exosome-particle aggregation following the storage for up to 7 days (Supplementary Figure S4). Note that the highest LC of 73.9% was almost unaffected, indicative for negligible release of ATS from EACs at 4°C within 7 days.

**TABLE 6:** Characterizations of mean size, zeta-potential and drug loading capacity of the studied Exo-ATS conjugates (EACs)^a^ that stored at 4°C during a 7-day observation period.

Storage time	Characterizations		
	Mean size (nm)	Zeta-potential (mV)	LC^b^ (mg ATS/100 mg exo-protein)
Day 0	68.74 ± 15.80^c^	−13.00 ± 1.60	73.9
Day 1	69.59 ± 16.46	−15.06 ± 1.23	74.8
Day 2	69.28 ± 16.08	−19.01 ± 0.41	75.1
Day 3	68.10 ± 15.58	−16.18 ± 0.72	75.1
Day 4	66.25 ± 14.52	−16.58 ± 1.44	75.0
Day 5	65.27 ± 14.09	−12.62 ± 0.58	75.4
Day 6	64.69 ± 13.58	−14.79 ± 0.41	75.3
Day 7	65.29 ± 13.66	−13.85 ± 0.35	75.4

^a^ The concentration of ATS, in EACs is 1.5 mg/mL (corresponding to EAC-3).

^b^ LC, loading capacity.

^c^ Data were presented as mean ± SD (*n* = 3).

The long-term (6-month) stability of EACs was further studied under various storage conditions (Supplementary Figure S5). As seen in Supplementary Figure S5A, the presence of a single peak at 9.051 min showed a good purity of EACs stored at 4°C for 1-month. With the extension of storage period to 3 months, however, a non-singular peak appeared at 11.634 min, potentially indicative for the release of impurity proteins from EACs. These suggested that the stability of EACs stored at 4°C could be maintained no longer than 3 months.

For each case of the EACs stored at −20°C and −80°C as well as the freeze-dried powder stored at room temperature, a single peak at around 9 min was seen in the purity analysis, representing a satisfactory purity similar to that shown in Figure 2E1. Despite a slight reduction in particle concentration of the EACs stored under different conditions along with the extension of storage time, no significant differences were found in mean size, zeta-potential and LC of EACs (Supplementary Figures S5B–E). Taken together, the results showed that the EACs stored at 4°C remained stable over 1-month. While the stability of EACs stored at −20°C and −80°C could be maintained over a 6-month period. And the freeze-dried powder of EACs obtained using lyophilization was able to be even stored at room temperature for over half a year.

### 3.4 Cellular uptake profile and ROS production of EACs *in vitro*

**FIGURE 4:**
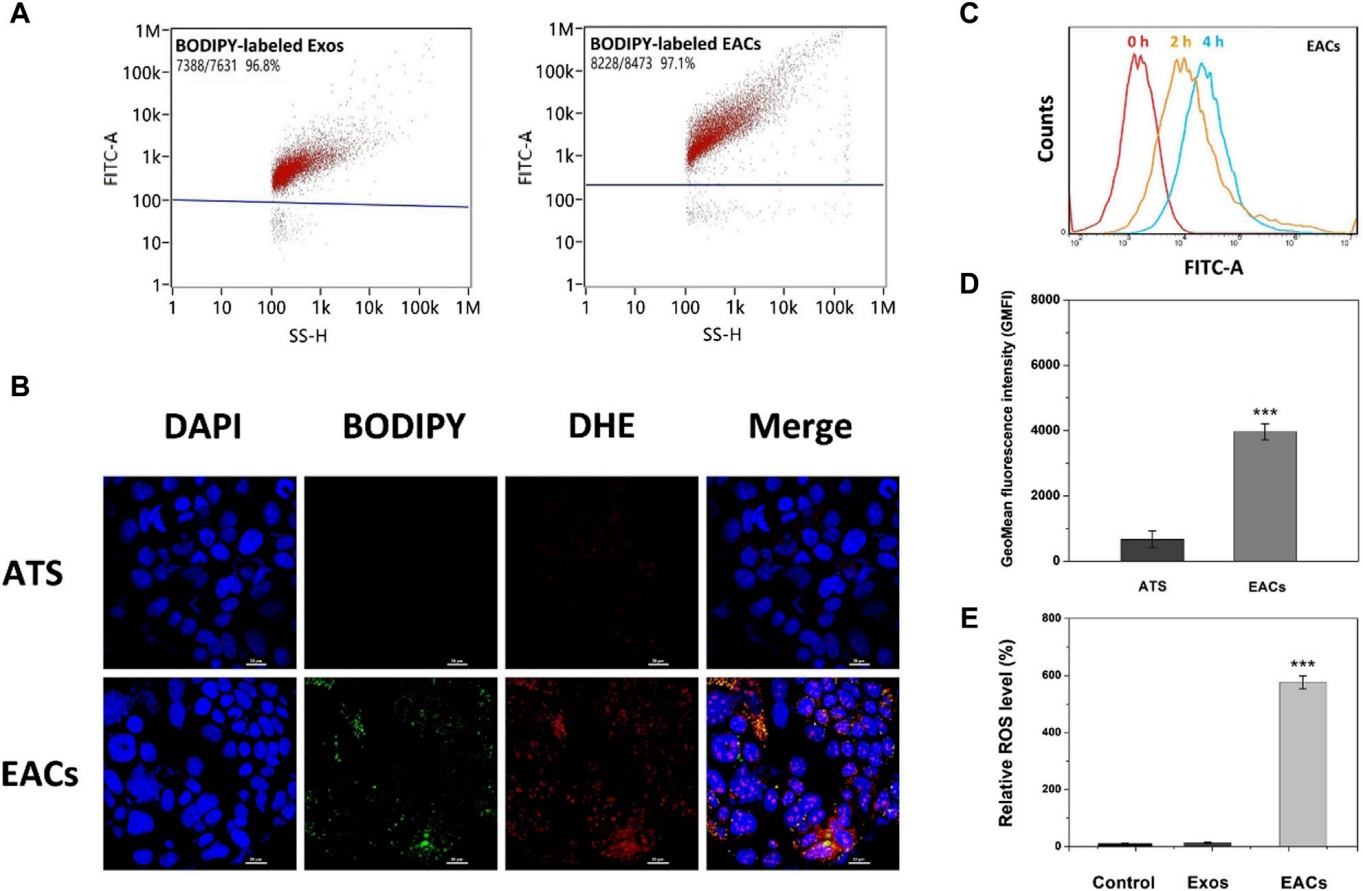
**(A)** Fluorescence labeling efficiency of BODIPY-labeled milk-derived exosomes (Exos) and Exo-ATS conjugates (EACs). **(B)** Cellular uptake profiles of ATS and EACs by Caco-2 cells at 4 h inspected by confocal laser scanning microscopy (CLSM) imaging. The nuclei of Caco-2 cells were stained by DAPI (blue) and the EACs were labeled with BPDIPY (green). The Reactive oxygen species (ROS) production induced by ATS (red) was shown by staining Caco-2 cells with 0.1 mM DHE after cellular uptake of ATS or EACs. Scale bar = 20 μm. **(C)** Flow cytometry analysis of the cellular uptake of BODIPY-labeled EACs by Caco-2 cells at 0, 2 and 4 h. **(D)** The geometrical mean fluorescence intensity (GMFI) detected by flow cytometry after the cellular uptake of ATS or BODIPY-labeled EACs by Caco-2 cells at 4 h. **(E)** Relative ROS level *in vitro* of the control group (saline), Exos and EACs (equivalent to 1.5 mg/mL ATS) after 4 h internalization by Caco-2 cells. Data were presented as mean ± SD, *n* = 3 (***p < 0.001 *versus* the ATS group). BODIPY = 2-(4,4-difluoro-5-methyl-4-bora-3a,4a-diaza-s-indacene-3-dodecanoyl)-1-hexadecaoyl-sn-glycero-3-phosphocholine, DAPI = 4′,6-diamidino-2-phenylindole, DHE = dihydroethidium.

Given that the exosomes derived from bovine milk were inherently able to traverse the intestinal barrier and thus readily absorbed intact from the intestine (Wolf et al., 2015; Agrawal et al., 2017), we investigated the cellular uptake of EACs using Caco-2 cell line so as to develop the exosomal formulation of ATS that exhibits improved oral bioavailability. In this work, the cellular uptake characteristics *in vitro* of EACs was profiled compared with free ATS at the same concentrations. Firstly, the EACs and unmodified Exos were labeled with BODIPY fluorescent probe. Figure 4A showed a fluorescence labeling efficiency of nearly 100% for both of Exos and EACs. This was indicative for an efficient labeling of EACs using BODIPY, which was almost unaffected by binding ATS to the Exos. The BODIPY-labeled EACs were then added to the Caco-2 cells for uptake evaluation.

In Figure 4B, we observed that the BODIPY-labeled EACs were internalized by Caco-2 cells after 4 h incubation, as shown by the nuclei (blue) surrounded by the clusters of EACs (green). Compared with free ATS, the remarkable green fluorescent signal from the BODIPY-labeled EACs led to an increased geometrical mean fluorescence intensity (GMFI) by 589.0% (*p* < 0.001) (Figure 4D). Given the fluorescence labeling efficiency of 97.1% for EACs, it showed that the enhanced GMFI was contributed by almost the entire EACs, implying a favored uptake profile of EACs by Caco-2 cells. In addition, we also tested the difference between the uptake efficiency of EACs and free ATS using HPLC determination of internalized ATS by Caco-2 cells. The data showed that the efficiency of ATS taken up by the cells increased from 5.7% to 84.5% by binding ATS to the Exos, confirming a more favored cellular uptake of EACs. This was in accordance with the findings with respect to the GMFI. These results demonstrated that EACs was able to improve the cellular uptake efficiency of ATS, indicating the good drug delivery ability of EDC system. Additionally, Figure 4C displayed an increased fluorescence intensity with the incubation time, showing that the EACs were internalized by Caco-2 cells in a time-dependent manner.

Due to the reactive oxygen species (ROS) originated from the rupture of the endoperoxide bridge of ATS (Zhang et al., 2015; Chen et al., 2019; Zhao et al., 2022), cellular uptake of EACs was further assessed by determination of intracellular ROS level. We tested the EAC-induced ROS generation compared with free ATS (equivalent to 1.5 mg/mL ATS) using dihydroethidium (DHE) probe after uptake by Caco-2 cells. In Figure 4B, the EACs-induced ROS generation was visualized (red), which showed a good co-localization with BODIPY-labeled EACs (orange and yellow). Instead, the produced ROS was hardly observed in ATS-treated cells. Such results confirmed again a preferred cellular internalization of EACs compared to free ATS by Caco-2 cells. In order to further quantify the EACs-induced ROS production, we determined the intracellular ROS level after the treatment with EACs *versus* ATS. As shown in Figures 4A, E few amount of ROS was produced in the groups of control (saline) and Exos, suggesting that the inherent background ROS level of the nature exosomes was negligible. Notably, EACs exhibited a significant enhancement in ROS generation by 572.5% (*p* < 0.001) in comparison with free ATS, showing again a higher internalization level of EACs by Caco-2 cells. The findings of ROS production agreed well with the cellular uptake profile.

### 3.5 Inhibition of EACs on cell proliferation

**FIGURE 5:**
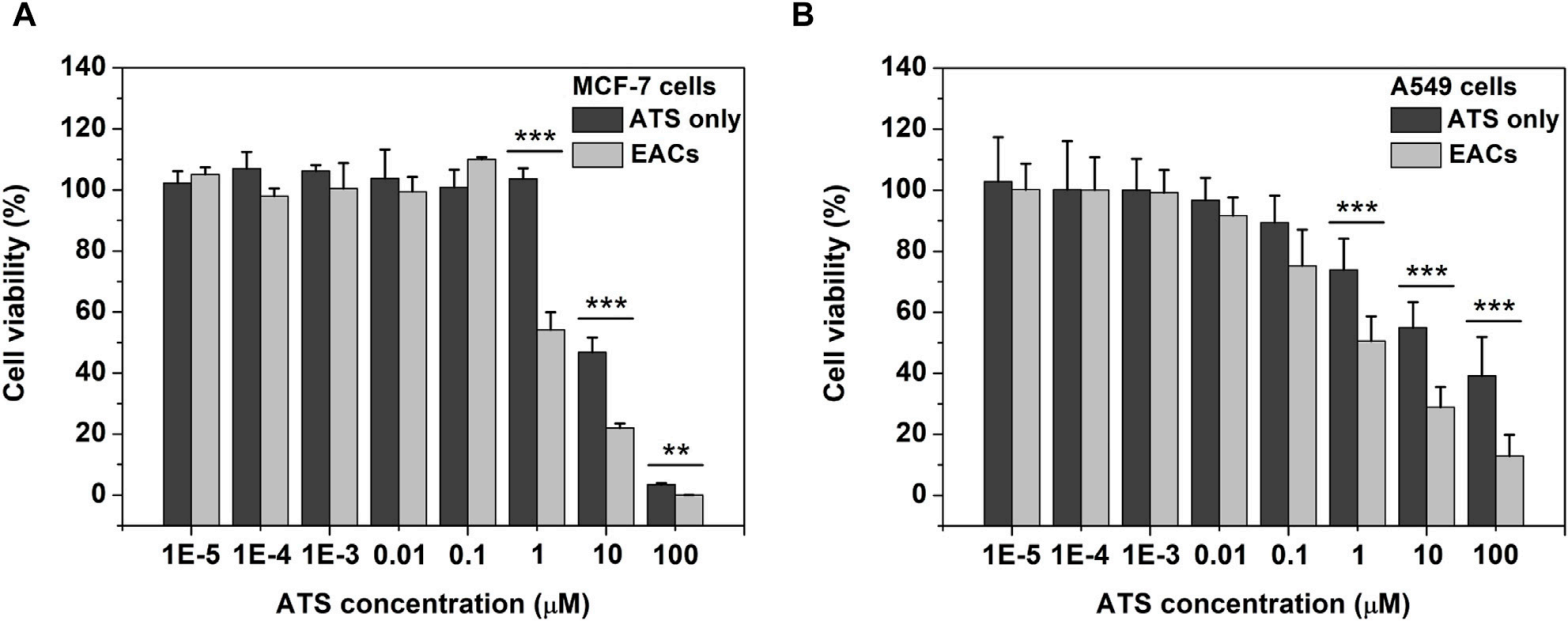
The cellular effect *in vitro* of Exo-ATS conjugates (EACs) and free ATS on the viability of **(A)** MCF-7 and **(B)** A549 cells after 48 h incubation. Data were presented as mean ± SD, *n* = 3 (**p < 0.01,***p < 0.001 *versus* the ATS group).

Given the remarkable ROS-productive capability of EACs, the cellular effect *in vitro* of EACs was next estimated using tumor cell lines including MCF-7 and A549 according to the earlier reports (Chen et al., 2019; Yang et al., 2021). As shown in Figure 5A, the viability of MCF-7 cells dramatically declined as the concentration of ATS increased from 1 to 100 μM, where the inhibited effect of EACs was higher than that of free ATS (*p* < 0.001 at 1 and 10 μM, *p* < 0.01 at 100 μM). In Figure 5B, compared with free ATS, EACs displayed similar inhibited effect on proliferation of A549 cells within 1–100 μM (*p* < 0.001). Combined with the cellular uptake profile, it could be deduced that the improvement in cytotoxicity *in vitro* of ATS against MCF-7 and A549 cells was ascribed to EACs delivery.

### 3.6 Release profile of ATS from EACs *in vitro*

**FIGURE 6:**
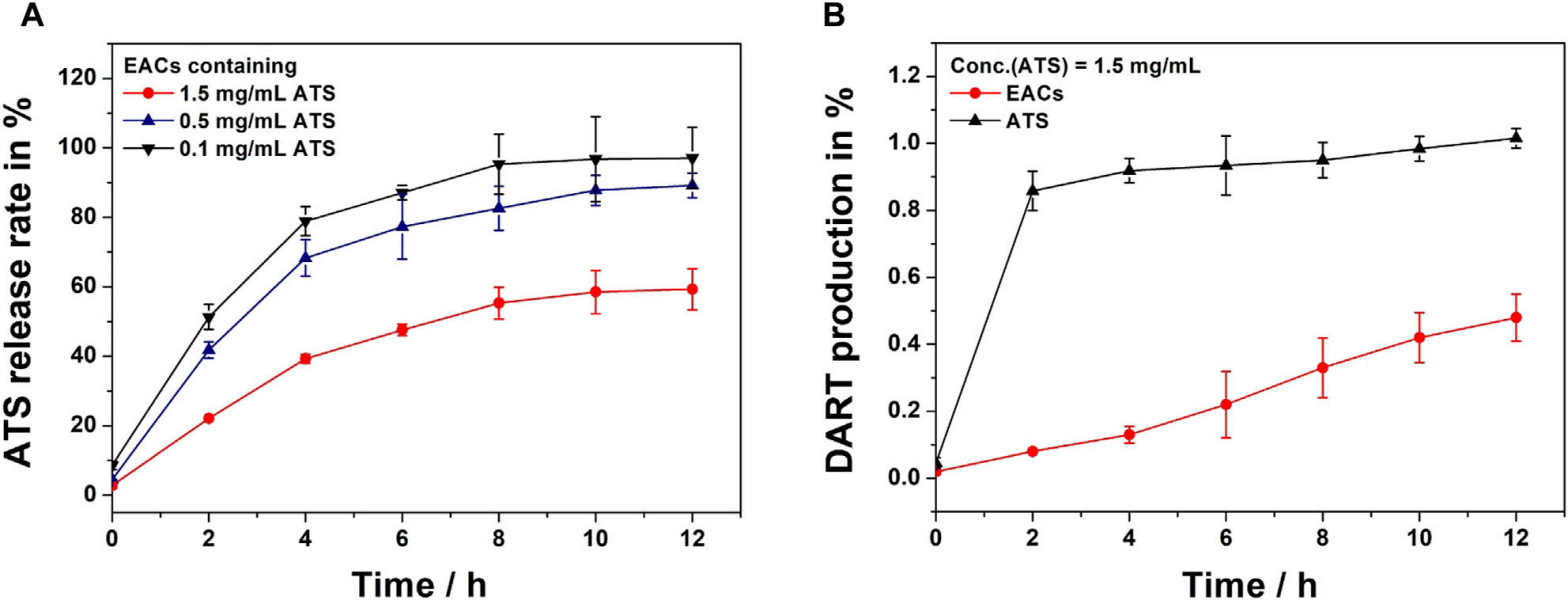
**(A)** *In vitro* release profiles of Exo-ATS conjugates (EACs) including EAC-1, EAC-2 and EAC-3 (equivalent to 0.1, 0.5 and 1.5 mg/mL artesunate (ATS), respectively) upon incubation in rat plasma at 37°C for 12 h. **(B)** Dihydroartemisinin (DART) production profiles of ATS and EACs (equivalent to 1.5 mg/mL ATS) upon incubation in rat plasma at 37°C for 12 h. Data were presented as mean ± SD (*n* = 3).

The release profiles *in vitro* of EAC-1, EAC-2 and EAC-3 (equivalent to 0.1, 0.5 and 1.5 mg/mL ATS, respectively) were shown in Figure 6A. We observed that ATS was released from each batch of EACs due to hydrolysis of the amide bond between ATS and Exos by proteases present in rat plasma. At 4 h, 78.9%, 68.3% and 39.3% of ATS were released from EAC-1, EAC-2 and EAC-3, respectively. Also, 95.3%, 82.6% and 55.3% of ATS were released from EAC-1, EAC-2 and EAC-3 at 8 h, respectively. And at 12 h, the release rate of ATS from EAC-1, EAC-2 and EAC-3 were 97.1%, 89.2% and 59.3%, respectively. The general release level of ATS from EACs negatively correlated with the concentration of ATS. EAC-1 and EAC-2 revealed similar release behavior and reached their maximal release levels of 97.1% and 89.2% at 12 h, respectively. By contrast, EAC-3 exhibited distinct sustained release profile.

Considering the degradation of ATS in rat plasma (Morris et al., 2011; Chinaeke et al., 2015; Zhang et al., 2018), the final active metabolite dihydroartemisinin (DART) of free ATS and EACs (equivalent to 1.5 mg/mL) was analyzed as well (Figure 6B). We found that ATS rapidly degraded into DART in rat plasma within 2 h as suggested by the DART production level of 85.8%. Conversely, less than 10% of DART were yielded by EACs. A gentle elevation in DART generation from EACs was observed and the yield level reached only 48% at 12 h. In contrast, ATS almost completely converted into DART at 12 h. These results showed that both of ATS and its active metabolite DART were yielded during the release from EACs into rat plasma. Therefore, both ATS and DART in plasma should be taken into account in the further pharmacokinetic (PK) profile of EACs.

### 3.7 Pharmacokinetic (PK) profile of EACs *in vivo*

**FIGURE 7:**
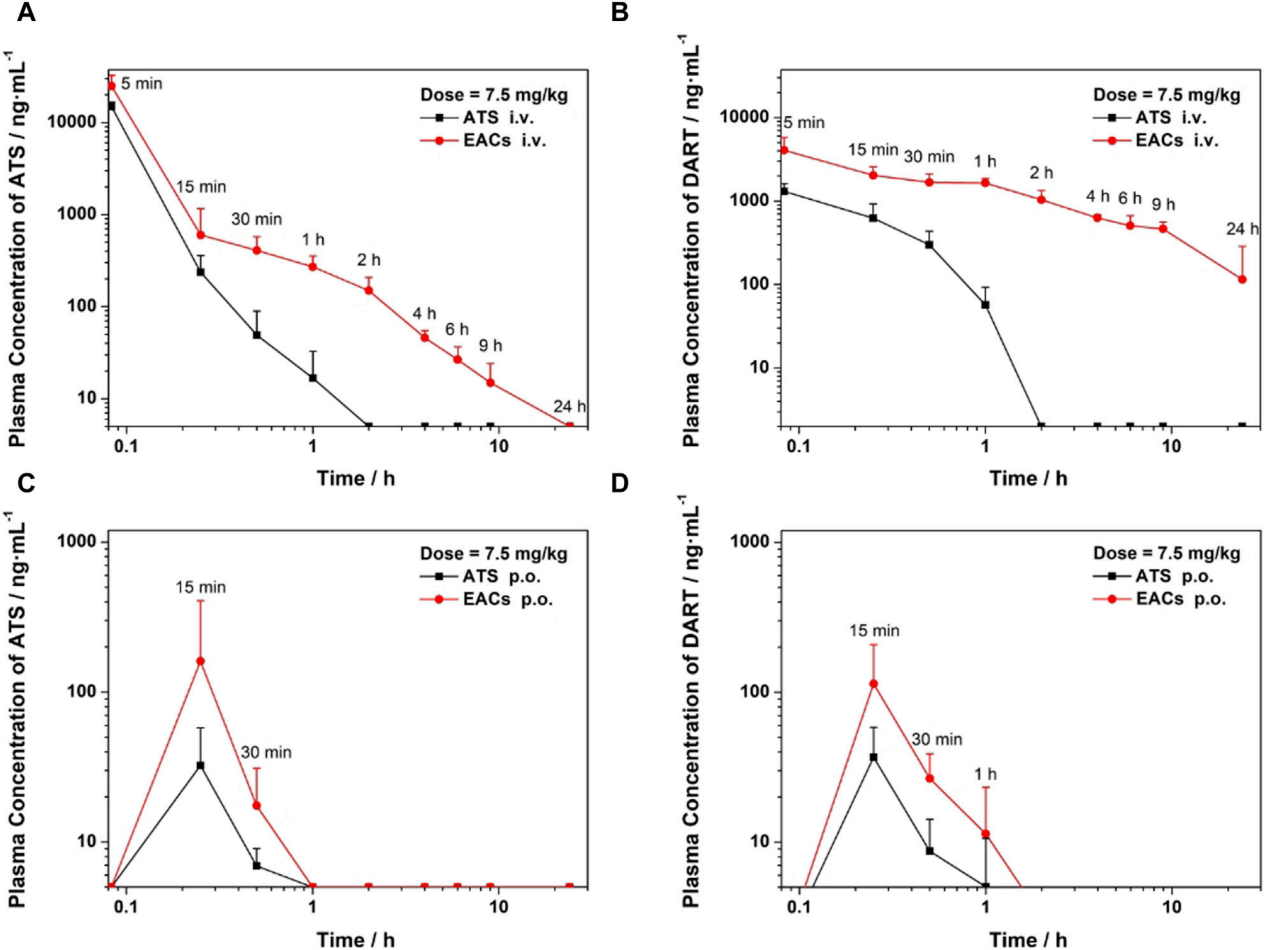
Pharmacokinetic profile *in vivo* of Exo-ATS conjugates (EACs) in comparison with artesunate (ATS) API for 24 h. Sprague-Dawley rats (6–8 weeks old, n = 16) were treated with a dosage of 7.5 mg/kg ATS, which were sub-divided into four groups: (i)/(ii) intravenous administration (i.v.) of EACs *versus* ATS API (equivalent to 1.5 mg/mL ATS) and (iii)/(iv) oral administration (p.o.) of EACs *versus* ATS API (equivalent to 0.75 mg/mL ATS). **(A, C)** Plasma concentration (i.v. and p. o.) of ATS plotted against time. **(B, D)** Plasma concentration (i.v. and p. o.) of the metabolite dihydroartemisinin (DART) of ATS plotted against time. Data were presented as mean ± SD (*n* = 4). API = active pharmaceutical ingredient.

Encouraged by the above *in vitro* results, we next profiled the pharmacokinetics (PK) of EACs via both oral and intravenous administration in comparison with the ATS API. This work was conducted using the Sprague-Dawley rat models administrated with either EACs or ATS API at the same dosage of 7.5 mg/kg for 24 h (Figure 7). Our study was thus sub-divided into four groups: (i)/(ii) intravenous administration (i.v.) of EACs *versus* ATS API (equivalent to 1.5 mg/mL ATS) and (iii)/(iv) oral administration (p.o.) of EACs *versus* ATS API (equivalent to 0.75 mg/mL ATS).

For the intravenous delivery of both the EACs and ATS API (Figure 7A), the peak plasma concentration (C_max_) appeared at 5 min after administration. And the ATS in both formulations was found to be completely eliminated in rat plasma within 24 h. Yet it was worth noting that the concentration of ATS API in rat plasma dramatically declined during the elimination phase and was not be detected at 2 h. By contrast, ATS in EACs was more gently eliminated in plasma, as suggested by its higher plasma concentration than that of ATS API at each sampling point as well as the complete elimination at 24 h. We calculated the key pharmacokinetic parameters including the area under curve (AUC) based on the detected the plasma concentration of ATS using UPLC, as shown in Table 7. The AUC for 24 h i. v. administration of EACs and ATS API were 12300 ± 4380 h·ng/mL and 7870 ± 1720 h·ng/mL respectively, indicating a 1.6-fold increase in AUC on average ascribed to the formulation of EACs. Moreover the half-life (t_1/2_) for the elimination phase of ATS in EACs was prolonged 27.6-fold compared to ATS API together with a 32.1% reduced clearance rate (CL). In addition, ATS in EACs exhibited a 8.1-fold increase in both the apparent volume of distribution (V_d_) and mean residence time (MRT) in comparison to ATS API. Such results profiled the pharmacokinetic behavior of EACs featured with improved bioavailability of ATS and sustained release of ATS from the EACs by i. v. administration.

**TABLE 7:** Pharmacokinetic (PK) profiles of Exo-ATS conjugates (EACs) and the artesunate (ATS) API for 24 h intravenous (i.v.) and oral (p.o.) administration of 7.5 mg/kg ATS (Corresponding to Figures 7A, C)^a^.

PK parameters^b^	ATS API^c^ (i.v.)	EACs (i.v.)	ATS API (p.o.)	EACs (p.o.)
T_max_ (h)	N/A^d^	N/A	0.25	0.25
C_max_ (ng/mL)	14900 ± 1709	24925 ± 7575	32.4 ± 25.2	161 ± 246
AUC_0-t_ (h·ng/mL)	7870 ± 1720	12300 ± 4380	37.1 ± 40.4	46.0 ± 67.9
AUC_0-∞_ (h·ng/mL)	7870 ± 1730	12300 ± 4380	N/A	N/A
t_1/2_ (h)	0.0943 ± 0.0563	2.6 ± 1.78	N/A	N/A
V_d_ (mL/kg)	22.7 ± 13.5	183 ± 131	N/A	N/A
CL (mL/h)	987 ± 211	670 ± 258	N/A	N/A
MRT_0-t_ (h)	0.0213 ± 0.00923	0.172 ± 0.082	0.7 ± 0.346	0.335 ± 0.0602
MRT_0-∞_ (h)	0.0216 ± 0.00898	0.254 ± 0.147	N/A	N/A

^a^ All the PK, parameters shown here were calculated based on the UPLC, determination of the rat plasma concentration of ATS., Data were presented as mean ± SD (*n* = 4).

^b^ C_max_ = peak plasma concentration, T_max_ = time to reach peak plasma concentration, AUC, area under the plasma concentration-time curve, t_1/2_ = half-life, V_d_ = apparent volume of distribution, CL, clearance rate; MRT, mean residence time.

^c^ API, active pharmaceutical ingredient.

^d^ N/A = not available.

As shown in Figure 7C and Table 7, the improvement in C_max_ of ATS by oral delivery of EACs suggested a favored absorption of ATS to some degree in comparison to ATS API (p.o.). Further, EACs revealed an 1.2-fold higher AUC than ATS API for 24 h oral administration. Given the findings of the presence of DART in rat plasma during the release of ATS from the EACs (Figure 6B), we hypothesized that the oral bioavailability of ATS was to some extent underestimated attribute to the degradation of ATS within 24 h. Consequently, the concentration of DART in rat plasma at each sampling time was again determined by UPLC and plotted against time in Figures 7B, D for intravenous and oral administration, respectively.

The plasma concentration-time curve of DART after i. v. administration of ATS API was present in Figure 7B, which got beyond that of ATS over the time range from 0.25 to 1 h. Moreover a gentle descending tendency was observed for the plasma concentration of DART compared to ATS API (Figure 7B). These results confirmed the degradation of ATS API to DART in rat plasma. Consequently, the pharmacokinetics of both the formulations of EACs and ATS API were re-profiled according to the detected concentration of DART in rat plasma (Table 8). Oral administration of EACs achieved a 3.1-fold elevation in C_max_ and a 2.6-fold improvement in bioavailability in comparison with oral delivery of ATS API. This finding manifested the compromised oral bioavailability of ATS due to the degradation of ATS to its metabolite DART in rat plasma and thus well supported our earlier hypothesis. For 24 h i. v. administration, the AUC of DART for the formulations of EACs and ATS API were 12899 ± 1993 h·ng/mL and 516 ± 108 h·ng/mL, respectively, indicative for a nearly 25-fold increase in AUC attribute to the EACs. Furthermore, EACs displayed a t_1/2_ of 7.28 ± 5.75 h and a CL of 545 ± 148 mL/h for DART during the elimination, which were 36.2-fold prolonged and nearly 96.4% decreased compared to the i. v. delivered ATS API respectively. These findings again confirmed the ameliorated bioavailability for both of p. o. and i. v. delivery of EACs and significant sustained-release action of ATS from the EACs via i. v. administration.

**TABLE 8:** Pharmacokinetic (PK) profiles of Exo-ATS conjugates (EACs) and the artesunate (ATS) API for 24 h intravenous (i.v.) and oral (p.o.) administration of 7.5 mg/kg ATS (Corresponding to Figures 7B, D)^a^.

PK parameters^b^	ATS API^c^ (i.v.)	EACs (i.v.)	ATS API (p.o.)	EACs (p.o.)
T_max_ (h)	N/A^d^	N/A	0.25	0.25
C_max_ (ng/mL)	1306 ± 274	4063 ± 1494	36.9 ± 18.6	114 ± 81.1
AUC_0-t_ (h·ng/mL)	516 ± 108	12899 ± 1993	18.4 ± 7.18	45.3 ± 32.3
AUC_0-∞_ (h·ng/mL)	524 ± 110	15338 ± 5881	22.8 ± 10.9	59.0 ± 30.7
t_1/2_ (h)	0.201 ± 0.0358	7.28 ± 5.75	1.74 ± 0.805	0.643 ± 0.108
V_d_ (mL/kg)	4119 ± 1045	4735 ± 1320	N/A	N/A
CL (mL/h)	15011 ± 3344	545 ± 148	N/A	N/A
MRT_0-t_ (h)	0.256 ± 0.0343	6.77 ± 1.35	0.761 ± 0.260	0.464 ± 0.080
MRT_0-∞_ (h)	0.273 ± 0.0278	10.6 ± 7.42	2.04 ± 0.583	0.668 ± 0.130

^a^ All the PK, parameters shown here were calculated based on the UPLC, determination of the rat plasma concentration of the active metabolite dihydroartemisinin (DART) of ATS., Data were presented as mean ± SD (*n* = 4).

^b^ C_max_ = peak plasma concentration, T_max_ = time to reach peak plasma concentration, AUC, area under the plasma concentration-time curve, t_1/2_ = half-life, V_d_ = apparent volume of distribution, CL, clearance rate; MRT, mean residence time.

^c^ API, active pharmaceutical ingredient.

^d^ N/A = not available.

## 4 Discussion

To date many ongoing efforts have been devoted to improving the solubility and bioavailability of artemisinin by chemical modification, leading to the creation of a variety of candidate derivatives including artesunate (ATS). Despite a slight increase in solubility of artemisinin due to the hydrophilic modification of the hemi-succinate group, the intestinal absorption was still insufficient and the resulting bioavailability were far from satisfactory (Wang et al., 2021; Yang et al., 2021). These drawbacks seriously hampered its further clinical application, particularly, as oral delivery. To overcome the above obstacles, we developed a new exosome-based drug vehicle for efficient loading and delivering ATS by bio-conjugation. Specifically, a simple and feasible bio-conjugation reaction was employed to form Exo-ATS conjugates (EACs) by binding ATS to the surface of exosomes via amide-linkage in between. Such conjugation leads to highly dispersion of ATS molecules in aqueous phase, which greatly contributes to the improvement in solubility of ATS. In this work, four formations of EACs (EAC-1∼4) were prepared and quantitatively characterized. The maximal concentration of 1.5 mg/mL of ATS in EACs was reached, corresponding to around 71.4-fold improvement in solubility of ATS (Table 4). Moreover, the entrapment efficiency (EE%) of ATS and the ATS loading capacities (LC) were found to be 90.3% and 73.9%, respectively, which was much better in comparison with the previously reported conventional approaches for drug loading (Table 5). Additionally, in order to figure out the mean number of the ATS molecules conjugated to a single exosome-particle, we have calculated the drug (ATS)-to-exosome ratio (DER) of the EACs (Table 4). The term ‘DER’ was referred in our work for the first time and could be used to evaluate the loading capacities of exosomes through bio-conjugation in future. Furthermore, EACs revealed the good storage stability at 4°C over a 1-month period.

To develop the exosomal formulation of ATS with improved bioavailability, the cellular uptake *in vitro* and pharmacokinetic profiles *in vivo* of EACs were investigated using the optimal batch of EACs (1.5 mg/mL ATS). Firstly, we profiled the cellular uptake of EACs using Caco-2 cells compared with free ATS under the same concentration. Confocal laser scanning microscopy (CLSM) and flow cytometry were employed for qualitative and quantitative analyses, respectively. EACs favoured a higher cellular uptake profile of ATS (Figures 4B, D) and were internalized in a time-dependent manner by Caco-2 cells (Figure 4C). Meanwhile, it demonstrated that EACs were able to improve the delivery efficiency of ATS. This showed the advantage of EDC strategy in drug delivery compared to the passive diffusion of drug molecules, indicative for an enhanced oral bioavailability of ATS by EACs. As for the internalization mechanism of EACs, when the EACs are taken by cells via the membrane fusion pathway, the fluorescence probe for labeling the EACs was supposed to more or less remain on the cell membrane (Li et al., 2022). Since the nearly 100% of EACs were labeled by fluorescence probe in this work and the green fluorescence was mainly distributed around the nuclei rather than on the cell membrane, the EACs more likely enter the cells via endocytosis rather than membrane fusion.

The release rate of ATS from the EACs was considered as a key factor associated with the therapeutic efficacy. Therefore, the release profiles of the EACs with varying concentrations of 0.1, 0.5 and 1.5 mg/mL ATS were evaluated in rat plasma (Figure 6A). The release level of ATS was found to be negatively correlated with the concentration of ATS and a sustained-release action of ATS was observed for the optimal batch of EACs (1.5 mg/mL ATS). Moreover we found that both of ATS and its active metabolite DART were yield during the release from the EACs (Figure 6B). Hence, the contribution of DART in plasma to the bioavailability of ATS in EACs was considered in the further pharmacokinetic investigation.

Based on the above positive findings, the pharmacokinetic behavior of EACs was profiled using rat models via both oral and intravenous administration in comparison with free ATS (API). For oral delivery, the C_max_ of ATS in EACs was improved compared to ATS API and the relative oral bioavailability was 1.2 (Table 7). As shown in Figure 7A and Table 7, a 1.6-fold increase in AUC on average was seen for intravenous delivery of EACs compared to ATS API. And the significant sustained-release action of ATS from EACs was shown, as suggested by a 27.6-fold extended half-life and a 32.1% declined clearance rate of ATS during the elimination phase. However, we noted that ATS API was dramatically eliminated in rat plasma within 2 h. Considering the esterase-mediated metabolism of ATS in rat plasma as well as the previous findings with respect to the release properties of EACs (Figure 6B), this prompted us to take into account the influence of the degradation of ATS on the pharmacokinetic profile of EACs. We hypothesized that the oral bioavailability of ATS in EACs within 24 h was underestimated to some degree due to the degradation of ATS to its metabolite DART. Consequently, the concentration of DART in rat plasma was again determined at each sampling point by UPLC and the pharmacokinetics for both oral and intravenous delivery of EACs were thus re-profiled. The results shown in Figures 7B, D well supported our earlier hypothesis. We found that a 3.1-fold elevation in C_max_ and a 2.6-fold improvement in bioavailability were achieved by oral delivery of EACs compared to ATS API. Furthermore, the sustained release profile of intravenous administrated EACs in Figure 7A was again confirmed by a 36.2-fold prolonged half-life and nearly 96.4% decreased clearance rate of the metabolite DART of ATS (Table 8). We believed that the sustained-release formulations of EACs have the great potential for application in malaria therapy in the future.

## 5 Conclusion

As a proof of concept, for the first time, we showed that our developed bio-conjugation approach greatly contributed to the efficient loading of ATS onto the milk-derived exosomes without affecting the physiological properties and biological functions of the exosomes. The synthesized Exo-ATS conjugates (EACs) not only ameliorated the solubility and bioavailability of ATS but also enabled a sustained-release profile of ATS from EACs. The best improvement in the solubility of ATS (approximately 71.4-fold) was achieved by EACs containing 1.5 mg/mL ATS. Furthermore, the EACs revealed a more favored cellular uptake profile by Caco-2 cells and higher delivery efficiency of ATS compared with free ATS. The ROS generation of EACs was validated as well, which was consistent with the findings of cellular internalization. Further, *in vivo* pharmacokinetic study manifested that a maximum 2.6-fold amelioration in oral bioavailability of ATS by EACs as well as a significant sustained-release action of ATS from EACs by intravenous administration. Taken together, the exosome-drug conjugation (EDC) could be a prospective strategy aimed at improving the pharmacokinetic profile of natural product-based drug candidates with poor solubility, low bioavailability and short half-life. We hoped that our work opened up a new avenue for the development of EDC delivery systems for further clinic application.

## Data Availability

The raw data supporting the conclusion of this article will be made available by the authors, without undue reservation.
