# Supplementary material for: Exosome-drug conjugates delivery: a promising strategy for ameliorating the pharmacokinetic profile of artesunate
Source: Front Bioeng Biotechnol. 2024 Aug 12;12:1437787. doi: 10.3389/fbioe.2024.1437787 (PMC11345266; doi:10.3389/fbioe.2024.1437787)
Supplement: Supplementary file 1 [file DataSheet1.docx]

**Supplementary Materials**

**of**

**Exosome-Drug Conjugates Delivery: a Promising Strategy for Ameliorating the Pharmacokinetic Profile of Artesunate**

*Da Wang ^a^, Yunfei Bai ^a^, Guogang Cheng ^a^, Shengqiang Shen ^a^, Gengwu Xiao ^a^, Demei Ma ^a^, Ganggang Zhao ^a^, Wei Chen ^a,b^, Tianshi Li ^c^, Litao Zhang ^d*^, Xiaohu Ge ^a,b*^*

*^a^ TINGO Exosomes Technology Co., Ltd, Tianjin, China*

*^b^ Exosome Origin (Shenzhen) Technology Co., Ltd, Shenzhen, China*

*^c^ Plastic & Cosmetic Surgery, Peking University Shenzhen Hospital, Guangdong, China*

*^d^Department of Dermatology, Tianjin Academy of Traditional Chinese Medicine Affiliated Hospital, Tianjin, China*

1. mail addresses:

[zhanglitao@medmail.com.cn](mailto:zhanglitao@medmail.com.cn) (^*^L. Zhang)

[gexiaohu@tingocell.com](mailto:gexiaohu@tingocell.com)(^*^X. Ge)

**Contents**

[1. Preparation and characterization of artesunate (ATS)-loaded exosomes 3](#_Toc169958404)

[2. UPLC determination of dihydroartemisinin (DART) in rat plasma 3](#_Toc169958405)

[3. Measurement of the aqueous solubility of artesunate (ATS) 4](#_Toc169958406)

[4. Supplementary Figures 6](#_Toc169958407)

[Figure S1. 6](#_Toc169958408)

[Figure S2. 6](#_Toc169958409)

[Figure S3. 7](#_Toc169958410)

[Figure S4. 7](#_Toc169958411)

[Figure S5. 8](#_Toc169958412)

# Preparation and characterization of artesunate (ATS)-loaded exosomes

For ATS loading into exosomes, 120 μL ATS solution in DMSO (36 mM) were first mixed with 750 μL milk-derived exosomes with a concentration of 10^12^ particles/mL in 1.0 mL PBS buffer. In order to compare with exosome-ATS conjugation, three different methods of drug loading were investigated, including co-incubation, electroporation and lipofectamine transfection. Concerning the co-incubation, the mixture of ATS and exosomes was co-incubated at 37 °C for 3.5 h with shaking. As for the electroporation method, the mixture of ATS and exosomes was mixed with electroporation buffer (30 mM sucrose, 0.6% (w/v) sodium chloride, pH 2) and then transferred into a chilled 0.4 cm electroporation cuvette. Afterwards, the mixture was electroporated at 0.7 kV in 5 ms intervals for 5 times using Eppendorf Eporator (Eppendorf AG, Hamburg, Germany), followed by incubation at 37 °C for 30 min to allow for recovery of the exosomal membrane as previously reported with slight modifications^1^. For the lipofectamine transfection, 120 μL ATS solution in DMSO (36 mM) were first mixed with 50 μL Lipofectamine^TM^ 3000 reagent (Invitrogen) and then incubated at 37 °C for 15 min. Afterwards, the mixture was added into 750 μL milk-derived exosomes with a concentration of 10^12^ particles/mL in 1.0 mL PBS buffer and then incubated at 37 °C for another 45 min.

Next, the produced ATS-loaded exosomes via the above described methods were ultra-centrifuged at 100,000 × g for 90 min at 4 °C by Optima L-80 XP ultra-centrifuge with rotor SW32Ti (Beckman Coulter). The resulting supernatant containing unbound ATS was collected and subsequently analyzed using HPLC system as described in 2.5.1. The loading capacity (LC) was calculated using the following formula:

$$\text{LC (mg ATS/100 mg Exo-protein)}=\frac{\text{M}_{\text{ATS， add}}-\text{M}_{\text{ATS, unbound}}}{\text{M}_{\text{Exosome, add}}}\times100$$

Where *M_ATS, add_* and *M_Exosome, add_* stand for the initial addition amount of ATS and total exosomal proteins for each investigated method, respectively,and *M_ATS, unbound_* the detected content of unbound ATS in the supernatant by HPLC.

# UPLC determination of dihydroartemisinin (DART) in rat plasma

The concentration of DART in rat plasma due to the degradation of artesunate (ATS) was quantified by ZORBAX UPLC system using a 4.5 × 50 mm XDB C18 column (particle size 5.0 μm) (Agilent). Verapamil was employed as internal standard for determining the plasma concentration of DART. The separation was carried out with a flow rate of 0.5 mL/min using a gradient liquid phase consisting of 10 mM ammonium acetate aqueous solution as mobile phase A containing 0.1% (v/v) formic acid and acetonitrile as mobile phase B containing 0.1% (v/v) formic acid. The gradient was formulated as follows:

| Time (min) | Mobile phase A (%) | Mobile phase B (%) |
| --- | --- | --- |
| 0.00 | 70 | 30 |
| 0.30 | 70 | 30 |
| 1.20 | 5 | 95 |
| 3.00 | 5 | 95 |
| 3.10 | 70 | 30 |
| 4.00 | 70 | 30 |

The retention time of DART was 1.78 min compared with 1.80 min of verapamil, which was monitored at 210 nm. After the UPLC determination of the plasma concentration of the samples as described in Section 2.11, 30 μL plasma sample was added to 500 μL ethyl acetate to re-extract the component of DART, followed by the introduction of 10 μL verapamil working solution into the mixture. After mixing for 10 min by vortex mixer, the mixture was centrifuged at 12,000 rpm at 4 °C for 15 min. Thus, 450 μL of the supernatant was transferred into another glass tube, followed by evaporated with the nitrogen stream at 60 °C. Afterwards, 50 μL of MPA and MPB reagents were added to the glass tube. After mixing for 3 min by vortex mixer, the mixed solution was centrifuged at 3,000 × g at 4 °C for 15 min. 5 μL of the resulted mixture was analyzed by the UPLC method as aforementioned. The rat plasma-DART concentration was calculated against a standard curve of DART, which was obtained using a series of standard plasma samples with varying concentrations of DART.

# Measurement of the aqueous solubility of artesunate (ATS)

The aqueous solubility of ATS was quantified by ZORBAX UPLC system using a 4.5 × 50 mm XDB C18 column (particle size 5.0 μm) (Agilent). Verapamil was employed as internal standard for quantification of ATS. The separation was carried out with a flow rate of 0.5 mL/min using a gradient liquid phase consisting of 1 mM ammonium formate-5%(v/v) acetonitrile-95%(v/v) water as mobile phase A containing 0.1% (v/v) formic acid and 50%(v/v) methanol-50%(v/v) acetonitrile as mobile phase B. The gradient was formulated as follows:

| Time (min) | Mobile phase A (%) | Mobile phase B (%) |
| --- | --- | --- |
| 0.00 | 35 | 65 |
| 2.00 | 5 | 95 |
| 5.00 | 5 | 95 |
| 5.10 | 35 | 65 |
| 6.00 | 35 | 65 |

The retention time of ATS was 2.28 min compared with 1.08 min of verapamil, which was monitored at 210 nm. A stock solution of ATS (10 mM) was prepared by sonification for 1 h at 37 °C. 8 μL of stock solution was added to 792 μL PBS buffer (100 mM, pH 7.4), followed by centrifugation at 1,000 rpm at room temperature for 1 h. After that, the mixture was centrifuged at 12,000 rpm at room temperature for another 10 min. Next, 100 μL of the resulted supernatant was added to 900 μL MeOH and thus mixed well by vortex mixer. Subsequently, the mixed sample was centrifuged at 12,000 rpm at room temperature for 5 min, resulting in a 10-fold diluted sample. This step was twice repeated to gain a 1,000-fold diluted sample. Afterwards, 50 μL verapamil working solution and 200 μL MilliQ water were added to 200 μL of the 1000-fold diluted sample and mixed well by vortex mixer. 2 μL of the resulted mixture was analyzed by the UPLC method as aforementioned.

# Supplementary Figures


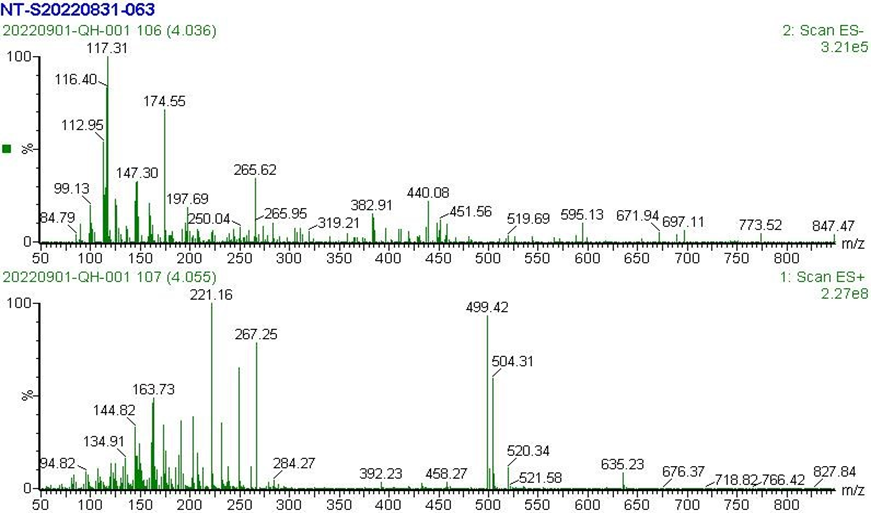


Figure S1. The MS spectrum of artesunate NHS ester.


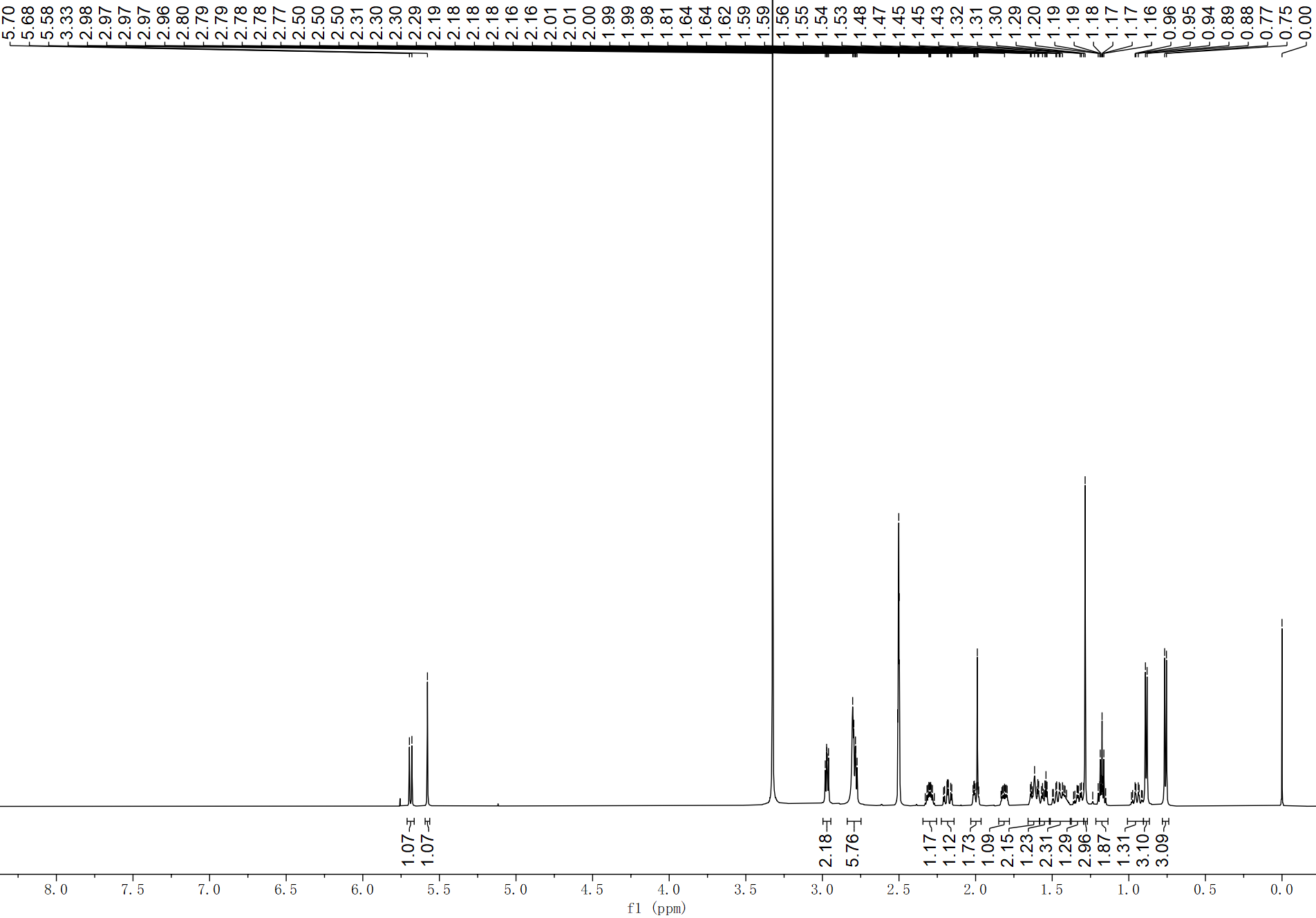


Figure S2. The ^1^H NMR spectrum of artesunate NHS ester (600 MHz, DMSO-*d_6_*).


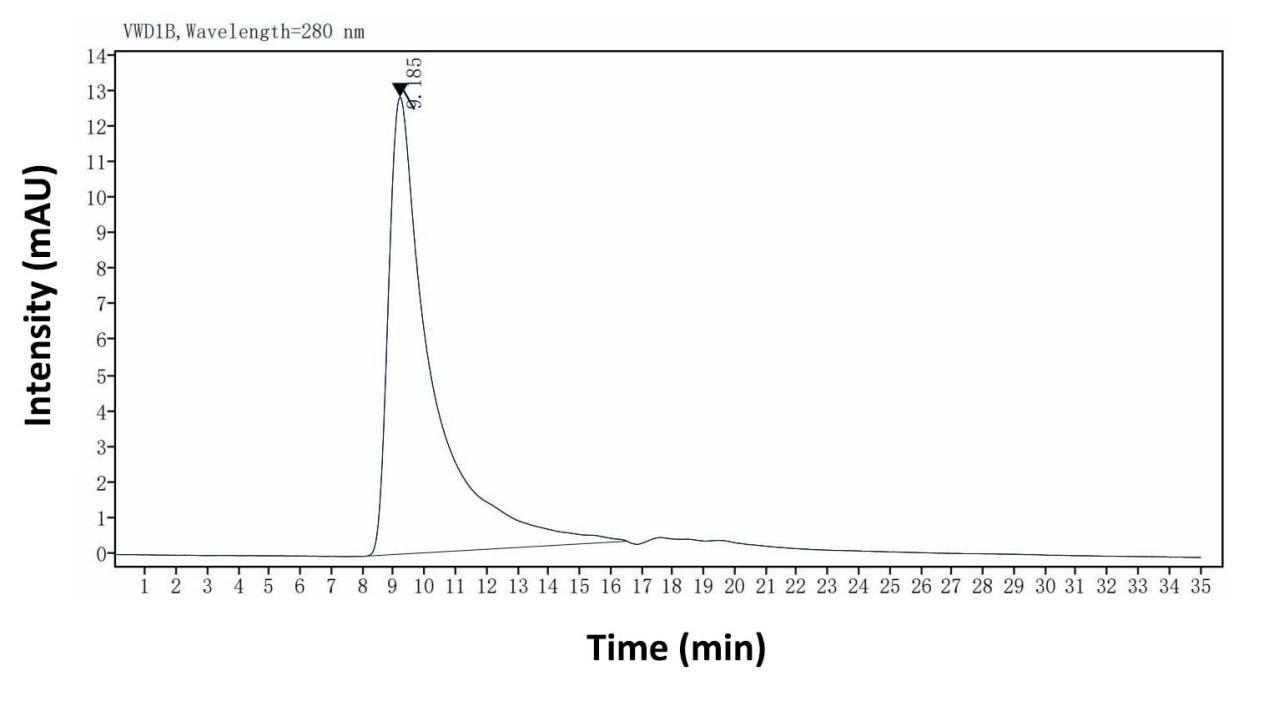


Figure S3. Identification of the exosomes derived from bovine milk by UPLC-SEC determination


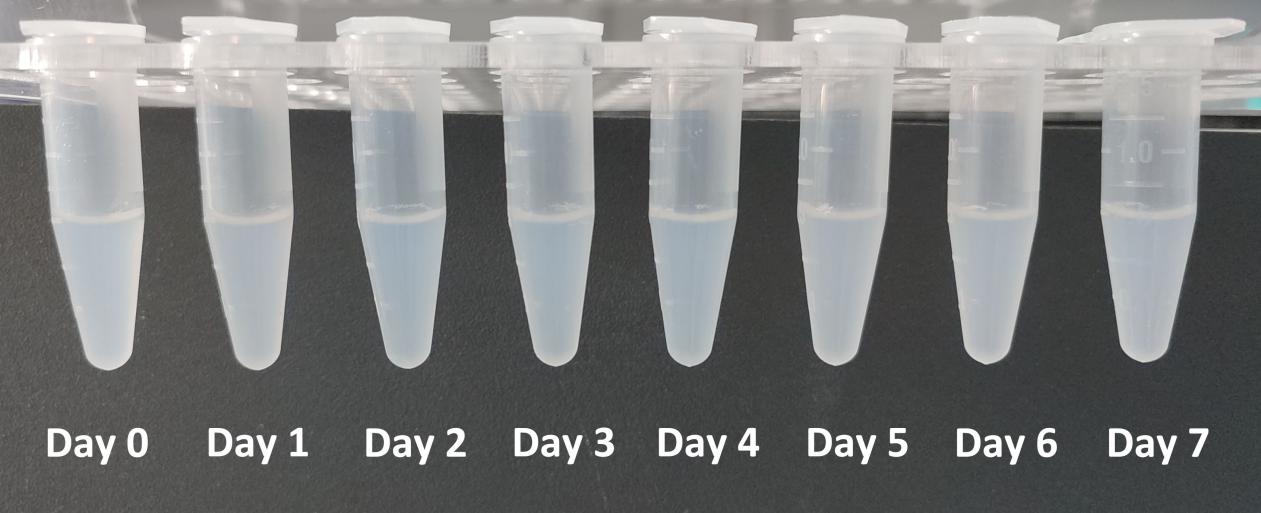


Figure S4. Physical appearance of the studied Exo-ATS conjugates (EACs, equivalent to 1.5 mg/mL ATS) following the storage at 4 °C for up to seven days (Day 0-7)

*
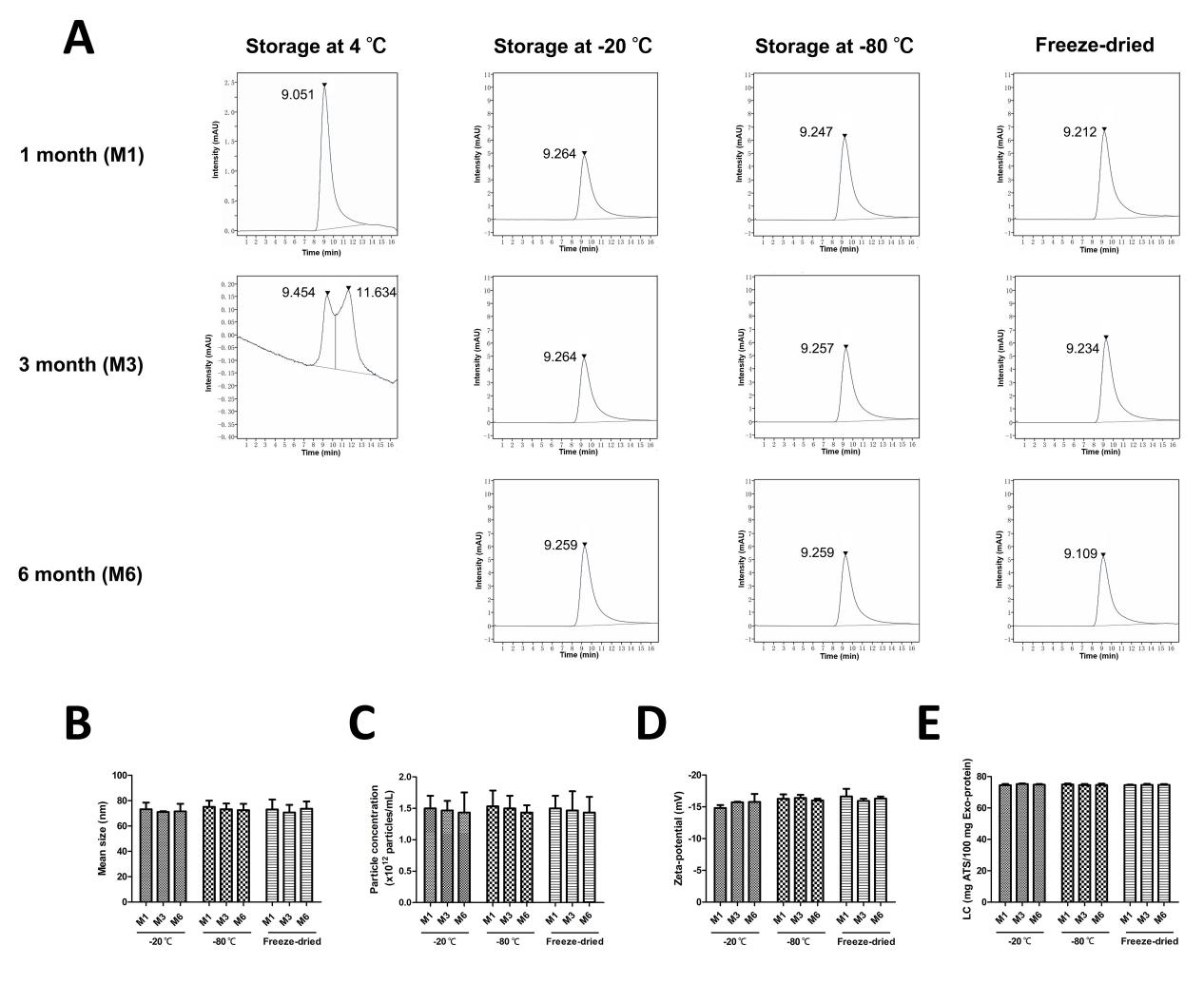
*

Figure S5. The long-term (six-month) stability of EACs stored under different conditions. (A) Purity analysis of EACs by UPLC-SEC. (B) Mean size and (C) particle concentration of EACs by NanoFCM analysis. (D) Zeta-potential of EACs measured by ZetaPALS. (E) ATS loading capacity (LC) of EACs determined by HPLC.

**References**

1. Kim M S, Haney M J, Zhao Y, et al. Development of exosome-encapsulated paclitaxel to overcomeMDR in cancer cells[J]. Nanomedicine: Nanotechnology, Biology and Medicine, 2016, **12**(3): 655-664.
